# A high stroma-tumor ratio is associated with an immunosuppressive tumor microenvironment and a poor prognosis in bladder cancer

**DOI:** 10.3389/fonc.2025.1604609

**Published:** 2025-08-22

**Authors:** Yiqiang Da, Zirong Lu, Zijian Zhu, Hongrui Tai, Yuan Liu, Yichao Zhu

**Affiliations:** ^1^ The First Clinical Medical College, Nanjing Medical University, Nanjing, Jiangsu, China; ^2^ School of Pediatrics, Nanjing Medical University, Nanjing, Jiangsu, China; ^3^ The Laboratory Center for Basic Medical Sciences, Nanjing Medical University, Nanjing, Jiangsu, China; ^4^ Department of Physiology, School of Basic Medical Sciences, Nanjing Medical University, Nanjing, Jiangsu, China; ^5^ Department of General Surgery, The Affiliated Taizhou People’s Hospital of Nanjing Medical University, Taizhou School of Clinical Medicine, Nanjing Medical University, Taizhou, Jiangsu, China

**Keywords:** stroma-tumor ratio, stromal score, bladder cancer, collagen, bioinformatics analysis

## Abstract

**Purpose:**

Bladder cancer (BLCA) is one of the most common urogenital malignancies in the world. The stroma of the tumor microenvironment (TME) largely affects the progression of BLCA. However, a stroma-relevant biomarker for predicting BLCA progression is still lacking.

**Methods:**

We obtained gene expression profiles and clinical data from the Cancer Genome Atlas (TCGA) datasets via UCSC Xena. The amount of stroma was evaluated using a stromal score and a stroma–tumor ratio (STR). The STR was independently assessed by two pathologists. The stromal score, derived from the R package “ESTIMATE,” was used to calculate the relative proportions of the stroma. We performed cell viability, wound healing, and Boyden chamber assays to determine cell behavior and utilized a BLCA in-house cohort to validate the results of our bioinformatics analysis.

**Results:**

Patients with a higher stromal content showed a worse prognosis. We found that to the high amount of stroma shaped a more immunosuppressive TME in BLCA. Next, we found that stroma could predict molecular subtypes and different therapy options in BLCA. A high stromal content shaped an immune overdrive TME. Cytological experiments revealed that collagen, the main component of the stroma, elevates BLCA cell viability, migration, and invasion. The results from the BLCA in-house cohort also showed that a high stromal content is associated with a worse prognosis and a higher PDL1 expression.

**Conclusion:**

A high stromal content shapes a more immunosuppressive tumor microenvironment and can predict not only the immune phenotypes but also the clinical phenotypes in BLCA. A high stromal content predicts a worse prognosis. STR exhibits great potential as a biomarker for evaluating the immunogenicity of BLCA and its likelihood of responding to immunotherapy.

## Introduction

Bladder cancer (BLCA) is one of the most aggressive malignant tumors in the world (1). In 2022, approximately 92,000 new cases of BLCA were reported in China (2). The tumor microenvironment (TME) is a complex and dynamic ecosystem, and its characteristics and composition have a significant impact on the treatment and prognosis of BLCA (3). Collagen, an essential component of the TME, has been shown to facilitate the extension, penetration, and invasion of tumor cells in BLCA by enhancing their adhesion and diffusion capabilities (4). The stromal score, derived from the R package “ESTIMATE,” serves as a metric to calculate the stromal content in the TME (5). At the histological level, we use the stroma–tumor ratio (STR) to represent the content of the stroma (6, 7). Moreover, the STR is independently assessed by two pathologists. Stromal cells are intimately associated with tumor growth, disease progression, and drug resistance (8). Typically, a high stromal content is associated with a greater stromal content within the tumor tissue (9). Wang et al. found that a high stromal content is correlated with poor prognosis in BLCA (10). However, research on the evaluation of the stroma in BLCA remains limited The underlying mechanisms by which an increased stromal content leads to a worse prognosis remain unknown. In this study, since collagen is the most important component of the TME (11), we used high collagen to imitate a high-stromal content TME (12). We also found that tumor cells showed higher invasion and migration in the collagen group, as proven in a cell experiment.

The relationship between the stroma and collagen-induced tumor cell migration and invasion was first proposed and experimentally validated in this study. We identified a strong connection between the stroma and an immunosuppressive TME. T-cell exhaustion occurs when T cells undergo chronic exposure to persistent antigens and sustained inflammatory stimulation in conditions such as prolonged infections or malignancies. This progressive dysfunction impairs their effector mechanisms, ultimately diminishing cytotoxic efficacy against neoplastic cells and compromising immune surveillance of pathological tissues (13). Bioinformatics analysis showed that T-cell exhaustion-related genes were highly expressed in the high stroma group, indicating that high tumor stroma may lead to the exhaustion of T cells in BLCA TME, as also revealed in our study. The majority of the immune factors were higher in the high stromalscore group. We also found that the stroma is closely associated with immunophenotypes, suggesting its potential to predict the molecular subtypes and the possibilities of various common therapies in BLCA. We found that tumors with higher stroma usually respond more positively to PD-L1 therapy. These outcomes, verified in the IMvigor210 and in-house cohorts, also confirmed our previous conclusion. In addition, we also found that higher stromal samples may lead to a TME-immune overdrive, thus explaining why high stroma tumors with higher immunity tended to have a worse prognosis. In summary, our findings demonstrate that the stroma is highly correlated with BLCA TME and clinical prognosis, highlighting the potential of STR as an efficient biomarker in BLCA.

## Materials and methods

### Data collection

Clinical information and transcriptional profiles from the TCGA-BLCA dataset were retrieved through the UCSC Xena platform (https://xenabrowser.net/datapages/). We obtained key information on signatures related to immunotherapy and other therapies from the link at the end of the article published in 2021 (http://www.thno.org/v11p3089s4.zip/) (14). Moreover, we retrieved the gene list of 133 immunomodulators from the article (15). To validate these molecular insights, we incorporated clinical data from the immunotherapeutic advanced urothelial cancer cohort (IMvigor210), a landmark study investigating immunotherapeutic interventions in advanced urothelial carcinoma, which was used to corroborate our findings, from http://research-pub.gene.com/IMvigor210CoreBiologies/ (16).

### Cell culture

The human BLCA cell lines 5637 (Cat.TCH-C104, HyCyte, Suzhou, China) and T24 (Cat.TCH-C352, HyCyte, Suzhou, China) were purchased from HyCyte (https://www.cas9x.com/). Specialized media for 5637 cells (Cat.TCH-C104, HyCyte, Suzhou, China) and T24 cells (Cat.TCH-C352, HyCyte, Suzhou, China) were used to maintain the BLCA cells. All cells were added with 10% FBS at 37°C in 5% CO_2_.

### Clinical samples

The BLCA tissue microarray (TMA, HBlaU079Su01) utilized in this study was procured from Outdo Biotech (Shanghai, China), comprising 63 neoplastic specimens and 16 matched adjacent normal tissue controls. The experimental protocols involving these histological samples obtained formal ethical clearance from the Institutional Review Board (Shanghai, China) of Outdo Biotech, in compliance with international biomedical research standards.

### Quantitative scoring

The STR was independently scored by two pathologists. All pathological sections were obtained from the website: https://cancer.digitalslidearchive.org/. For the assessment of different tumor cells, the stroma–tumor ratio was scored as follows: 0 (0%–50%) and 1 (50%–100%) (7).

### Survival analysis

The prognostic value of the stromal score for overall survival (OS) and disease-free survival (DFS) was analyzed using the bioinformatics website (http://www.sxdyc.com/) (17). The outcome of the Kaplan–Meier (KM) curve was presented with clinical data from the TCGA-BLCA and in-house cohorts (18).

### Estimation of the immunological characteristics of the TME

Stromal score, tumor purity, immune score, and ESTIMATE score were calculated using the ESTIMATE R package (19). This is why we divided all the samples equally into the high-stromal score group and the low-stromal score group according to the value of the stromal score (20). We estimated the immunological characteristics of the TME for each patient. Information on 133 immunomodulators, such as chemokines, immunoinhibitors, immunostimulators, Major Histocompatibility Complex (MHC), and receptors, was collected from previous studies (21). We also collected well-known effector genes of tumor-infiltrating immune cells (TIICs) to finish our bioinformatics analysis (22). To mitigate the potential miscalculations arising from various algorithms when estimating the levels of TIICs, we conducted a comprehensive analysis of their relative abundance utilizing the following algorithms: TIMER (23), EPIC (24), MCP-counter (25), and quanTIseq (26).

### Enrichment scores of different gene signatures

To graphically delineate oncogenic signaling cascades within the inflammatory tumor microenvironment, along with therapeutic responses to molecularly targeted agents and immune checkpoint modulation, a pathway enrichment analysis was conducted based on established computational frameworks from our prior investigations (27). Hypoxia (28), angiogenesis (29), and Epithelial - Mesenchymal Transition Score (EMT score) scores (30) were all calculated using the R package “GSVA.” All the gene sets can be found on the website: https://www.gsea-msigdb.org/. All enrichment scores of these signatures were calculated by the “GSVA” algorithm (31).

### Bioinformatics analysis

The analysis results of heatmap plots were generated using the CNSknowall platform (https://cnsknowall.com/). Furthermore, we also applied the R version 4.3.3 to finish several bioinformatics plots, such as violin plots and triangle plots. In addition, bioinformatics analyses were performed using tools in Hiplot Pro (https://hiplot.com.cn/), a web-based tool for bioinformatics analysis and visualization (32).

### Cell viability assay (CCK-8 assay)

Cell viability was quantitatively assessed using the CCK-8 methodology with 96-well microplates. The experimental cohorts included the 5637 and T24 urothelial carcinoma cell lines, with the test groups receiving collagen treatment, and the control groups maintaining negative control (NC) conditions under standardized culture protocols (33). We prepared the acetic acid solution (68.8 μL of glacial acetic acid + 40 mL of double-distilled water) and then the collagen stock solution (100 μg/mL) (collagen: acetic acid solution = 1:30). After incubation at 37°C for 24 h, 10 µL of the CCK-8 reagent was added to each well, and this was cultured continuously for 1 h. Optical density (OD) was detected by a microplate reader Thermo Fisher Scientific Multiskan™ FC Microplate Photometer(Cat.1410101, Shanghai, China).

### Wound healing assay

The 5637 and T24 cells were seeded in a six-well cell culture plate. Once the cells reached confluence, sterile tips were employed to create scratches in the cell monolayer within the six-well plate (34). We prepared the acetic acid solution (68.8 μL of glacial acetic acid + 40 mL of double-distilled water) and then the collagen stock solution (100 μg/mL) (collagen:acetic acid solution = 1:30). Following a 24-h incubation under physiological conditions (37°C), cellular migration dynamics were systematically monitored through time-lapse imaging at 0- and 12-h intervals. Quantitative analysis of wound closure was performed by calculating the mean migration distance across three distinct microscopic fields per scratch boundary using phase-contrast microscopy (Mshot M152-N, Guangzhou, China) with a magnification of ×100.

### Boyden chamber assay (invasion assay)

Cell invasion potential was evaluated through a modified Boyden chamber assay utilizing 24-well inserts pre-coated with growth factor-reduced Matrigel matrix (30 μL, 1:1 dilution in serum-free medium, Cat. 356234, Corning, Bedford, MA, USA). The Transwell apparatus contained polycarbonate membranes with 8.0-μm porosity (Corning, USA), following standardized *in vitro* invasion protocols (35). We prepared the acetic acid solution (68.8 μL of glacial acetic acid + 40 mL of double-distilled water) and then the collagen stock solution (100 μg/mL) (collagen:acetic acid solution = 1:30). After a standard 24-h culture under physiological conditions (37°C, 5% CO_2_), residual cells remaining on the apical aspect of the polycarbonate membranes were mechanically removed with sterile cytology swabs. The migratory cell populations that successfully traversed the matrix barrier underwent methanol fixation followed by morphological visualization using 0.1% crystal violet solution (in 2% ethanol). Quantitative assessment was achieved through the systematic enumeration of stained cells across five predefined microscopic fields per chamber using phase-contrast microscopy (Mshot M152-N, Guangzhou, China), with data normalized to control conditions.

### Statistical analysis

Quantitative analyses were implemented through GraphPad Prism 8.0 with rigorous statistical validation. Intergroup variations were determined via parametric one-way ANOVA, complemented by Bonferroni-corrected *post-hoc* examinations to address type I error inflation in multigroup comparative analyses. An unpaired, two-tailed Student’s *t*-test was used for the comparison of two groups. Correlation analyses were performed using Pearson’s correlation coefficient. Kaplan–Meier (KM) analyses were performed using the log-rank test. For all analyses, a two-tailed *P* <0.05 was considered statistically significant, unless otherwise indicated, unless otherwise indicated. Statistical significance was defined as *P < 0.05, **P < 0.01, ***P < 0.001, and ****P < 0.0001 (36).

## Results

### A high stromal content predicts a poor prognosis outcome

As one of the most important components of the TME, the tumor stroma influences tumor biology by promoting tumor initiation, progression, metastasis, and treatment resistance (37). The tumor stroma manifests distinct spatiotemporal dynamics, architectural heterogeneity, and context-dependent molecular signatures. This pathologically specialized niche comprises two distinct compartments: (1) acellular constituents featuring remodeled extracellular matrix (ECM) architectures and aberrant neovascular networks, and (2) cellular constituents encompassing activated fibroblastic lineages (CAFs), multipotent mesenchymal progenitors, perivascular support cells, and immunomodulatory elements (38). The stromal score was derived using the ESTIMATE algorithm, which computes tumor stromal content.

**Figure 1:**
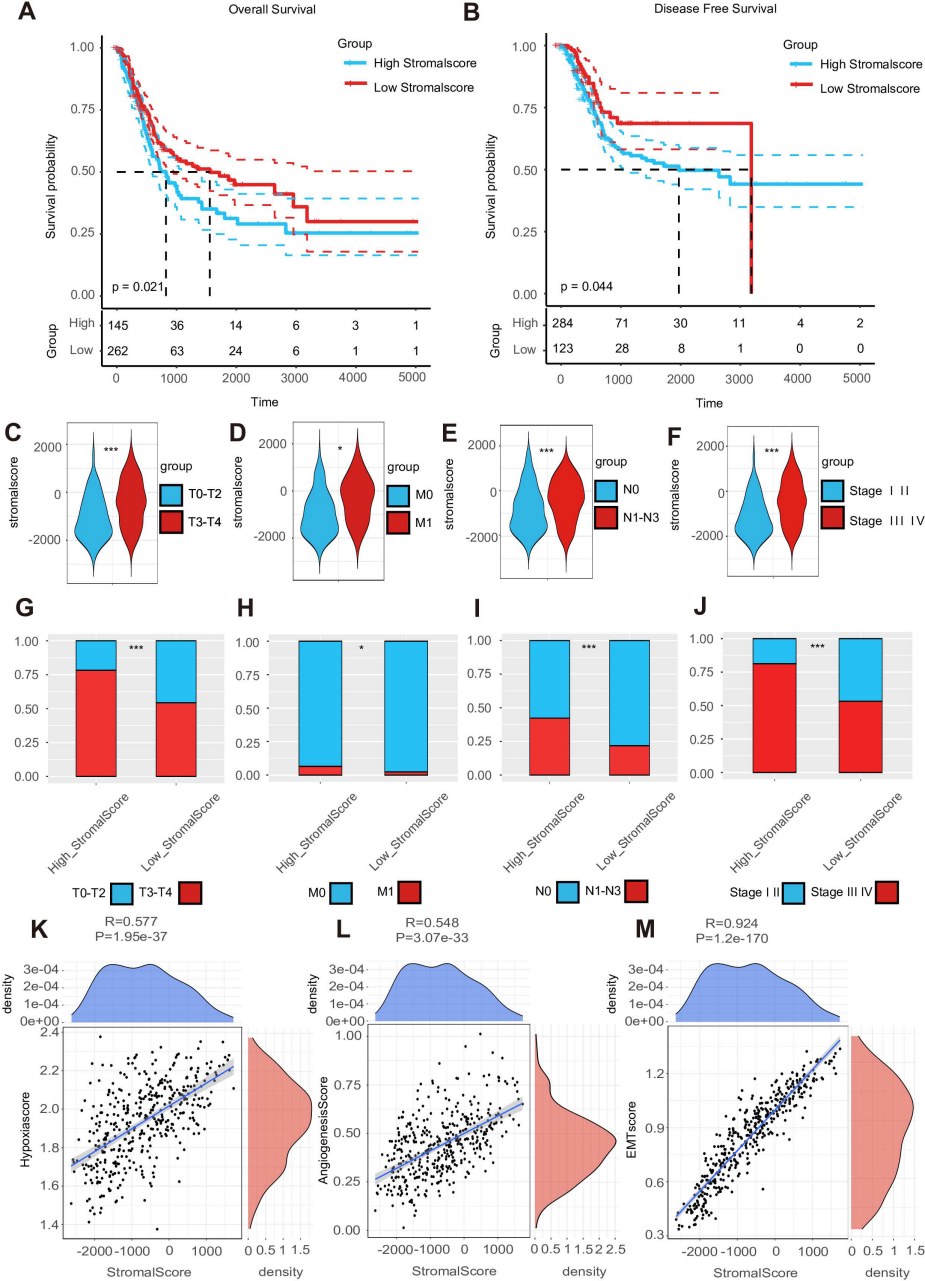
A high stroma predicts a poor prognosis outcome. **(A, B)** The overall survival (OS) Kaplan–Meier plot and disease-free survival (DFS) Kaplan–Meier plot for subgroups with different stromal scores. **(C–F)** Expression of the stromal score in subgroups with different clinical characteristics. **(G–J)** A double-group bar chart of patients with different clinical characteristics in subgroups with different stromal scores. **(K–M)** Correlation plots among stromal, hypoxia, angiogenesis, and EMT scores.

Using the median stromal score as a stratification threshold, BLCA patients were categorized into high-stromal score and low-stromal score groups. A comparative analysis of clinicopathological parameters revealed significant intergroup disparities in survival status across both the TCGA-BLCA dataset and our in-house cohort. This stratification methodology effectively differentiated patient populations with distinct prognostic profiles (**Table 1**). The Kaplan–Meier overall survival analysis based on the optimal cutoff value showed that the low-stromal score group showed better OS compared with the high-stromal score group, which represented better prognosis outcomes (**Figure 1A**) (17, 39). The DFS Kaplan–Meier plot based on the optimal cutoff value demonstrated that the high-stromal score group showed a poorer survival probability than the low-stromal score group, also supporting the association with worse prognosis outcomes (**Figure 1B**) (17, 39). Next, we stratified patients into subgroups based on T stage (T0–2 to T3–4), N stage (N0 to N1–3), M stage (M0 to M1), and AJCC stage (stages I–II to stages III–IV). According to the violin plots, STRs varied significantly across these subgroups, suggesting a strong correlation between a high stromal score and a poor prognosis. Specifically, higher stromal scores were observed in the T3–4, N1–3, M1, and stage III–IV subgroups (**Figures 1C–F**). We calculated the number of tumor samples in the high-stromal score group and the low-stromal score group according to the different stages, including T stage, N stage, M stage, and AJCC stage (**Figures 1G–J**). The histograms of the different stages showed that the high-stromal score group tended to manifest worse stages, such as T3–T4 stage, M1 stage, N1–N3 stage, and stage III–IV, which are usually associated with a worse prognosis. In addition, hypoxia score (28), angiogenesis score (29), and EMT score (30) are usually associated with poor prognosis in tumors. We also made a correlation plot between the stromal score and the hypoxia score (**Figure 1K**), angiogenesis score (**Figure 1L**), and EMT score (**Figure 1M**), which indicated that a high stromal score was correlated with a worse prognosis.

**Table 1:** Clinicopathological features between the two clusters identified by the STR from the TCGA-BLCA and in-house cohorts.

CharacteristicsSTR	High STR (*N* = 234)	Low STR (*N* = 234)	*P*-value
Gender			0.39
Female	62 (26.5%)	53 (22.6%)	
Male	172 (73.5%)	181 (77.4%)	
Age			0.138
≤60	52 (22.2%)	68 (29.1%)	
>60	182 (77.8%)	165 (70.5%)	
Unknown	0 (0%)	1 (0.4%)	
T stage			2.3e−11
T0–T2	51 (21.8%)	102 (43.6%)	
T3–T4	175 (74.8%)	102 (43.6%)	
Unknown	8 (3.4%)	30 (12.8%)	
N stage			1.2e–07
N0	131 (56.0%)	149 (63.7%)	
N1–N3	91 (38.9%)	45 (19.2%)	
Unknown	12 (5.1%)	40 (17.1%)	
Stage			4.4e−11
Stage 0–II	45 (19.2%)	109 (46.6%)	
Stage III–IV	187 (79.9%)	116 (49.6%)	
Unknown	2 (0.9%)	9 (3.8%)	

We performed pathological section scoring to assess the stroma–tumor ratio. All pathological sections were obtained from the Cancer Digital Slide Archive (https://cancer.digitalslidearchive.org/). We found that a high-stromal score sample was usually related to a high STR sample under the *t*-test result (**Supplementary Figure S2A**). The average stromal score in the high-STR group was approximately 404.8044, while in the low-STR group, it was only −1,166.3234. The stroma–tumor ratio was scored as follows: 0 (0%–50%) and 1 (50%–100%). Those samples with high STRs were usually scored as 1, such as the TCGA-DK-A3IQ (**Supplementary Figure S1A**), TCGA-FD-A5BS (**Supplementary Figure S1B**), TCGA-XF-AAME (**Supplementary Figure S1C**), and TCGA-FD-A5BT samples (**Supplementary Figure S1D**). On the contrary, samples with low STRs were scored as 0, as seen with the TCGA-ZF-AA4X (**Supplementary Figure S1E**), TCGA-ZF-A9RM (**Supplementary Figure S1F**), TCGA-E7-A5KF (**Supplementary Figure S1G**), and TCGA-ZF-AA4U samples (**Supplementary Figure S1H**). In summary, a high stroma was associated with poor survival prognosis outcomes.

### A high stroma is closely linked with an immunosuppressive TME

**Figure 2:**
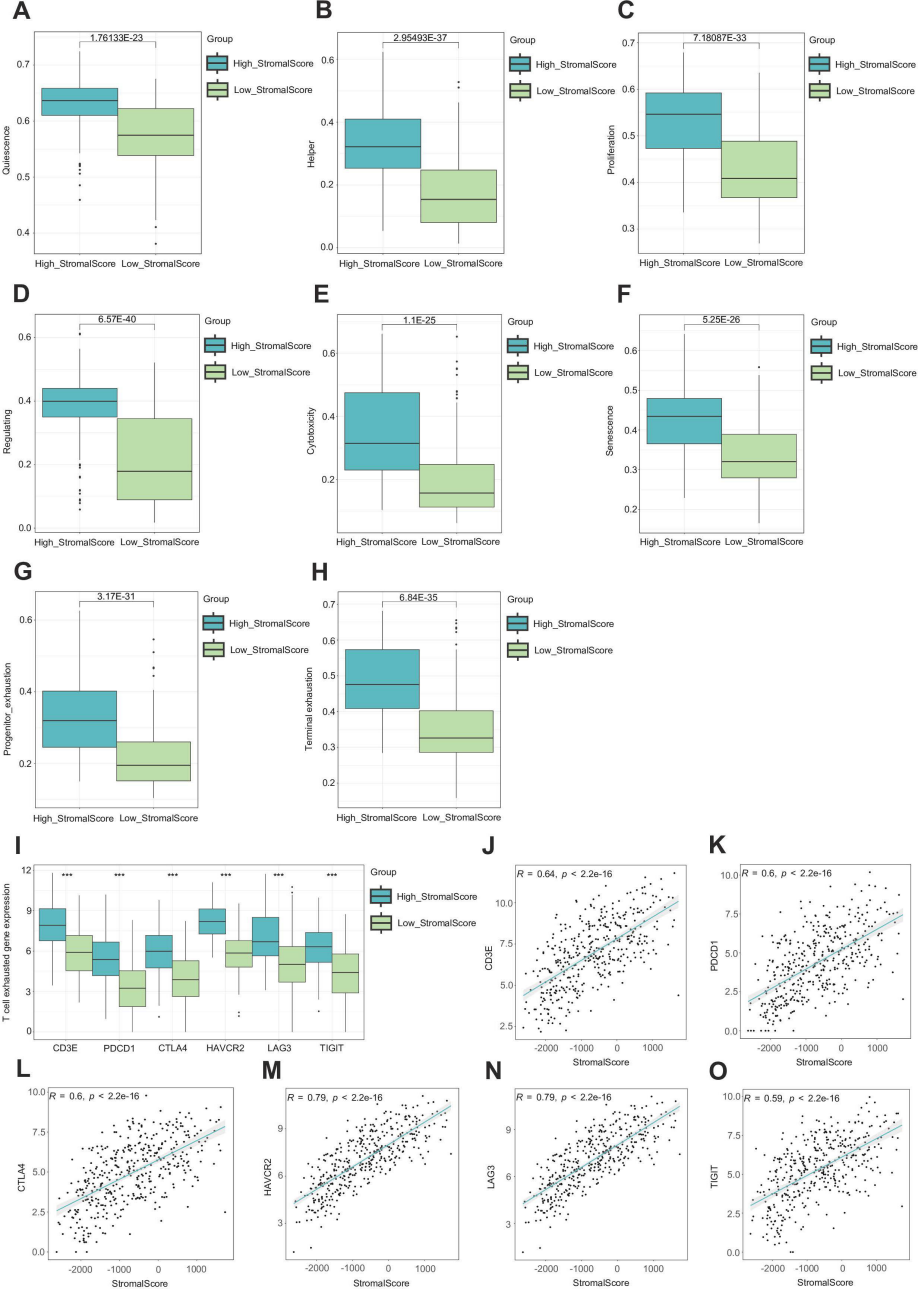
A high stroma leads to T cell exhaustion. **(A–H)** Evaluation of eight different states of T cells in subgroups with different stromal scores. **(I)** Expression of CD3E, PDCD1, CTLA4, HAVCR2, LAG-3, and TIGIT in subgroups with different stromal scores. **(J–O)** Correlation plots between the expression of CD3E, PDCD1, CTLA4, HAVCR2, LAG-3, TIGIT, and stromal score.

Because the stroma is so important to the TME, we conducted research on the function of tumor stroma in shaping the BLCA immune microenvironment. Comprehensive phenotypic profiling of eight functionally distinct T-cell subsets (quiescent, regulatory, proliferating, helper, cytotoxic, progenitor-exhausted, terminally exhausted, and senescent populations) was performed across stromal score-stratified subgroups. Comparative analysis demonstrated significantly elevated activation scores across all T-cell compartments in high-stromal score cohorts, with particularly pronounced enrichment observed in both progenitor-exhausted and terminally exhausted subsets. These differential activation patterns suggest potential mechanistic links between a high stroma and T-cell exhaustion progression (**Figures 2A–H**). As we all know, the T cell is an important immune cell in the TME. We hypothesized that a richer tumor stroma could exhaust T cells, thus resulting in a worse prognosis (9). We searched for information and found that several genes are related to T-cell exhaustion, such as CD3E, PDCD1, CTLA4, HAVCR2, LAG-3, and TIGIT (40, 43). Bioinformatics analysis revealed significantly elevated expression levels of these candidate genes in the high-stromal score cohort (**Figure 2I**). Subsequent correlation analysis demonstrated consistent positive associations between stromal scores and the specified T-cell exhaustion marker. Importantly, these patterns suggest that a high stroma may drive immunosuppressive microenvironment remodeling through T-cell exhaustion (**Figures 2J–O**) (41).

**Figure 3:**
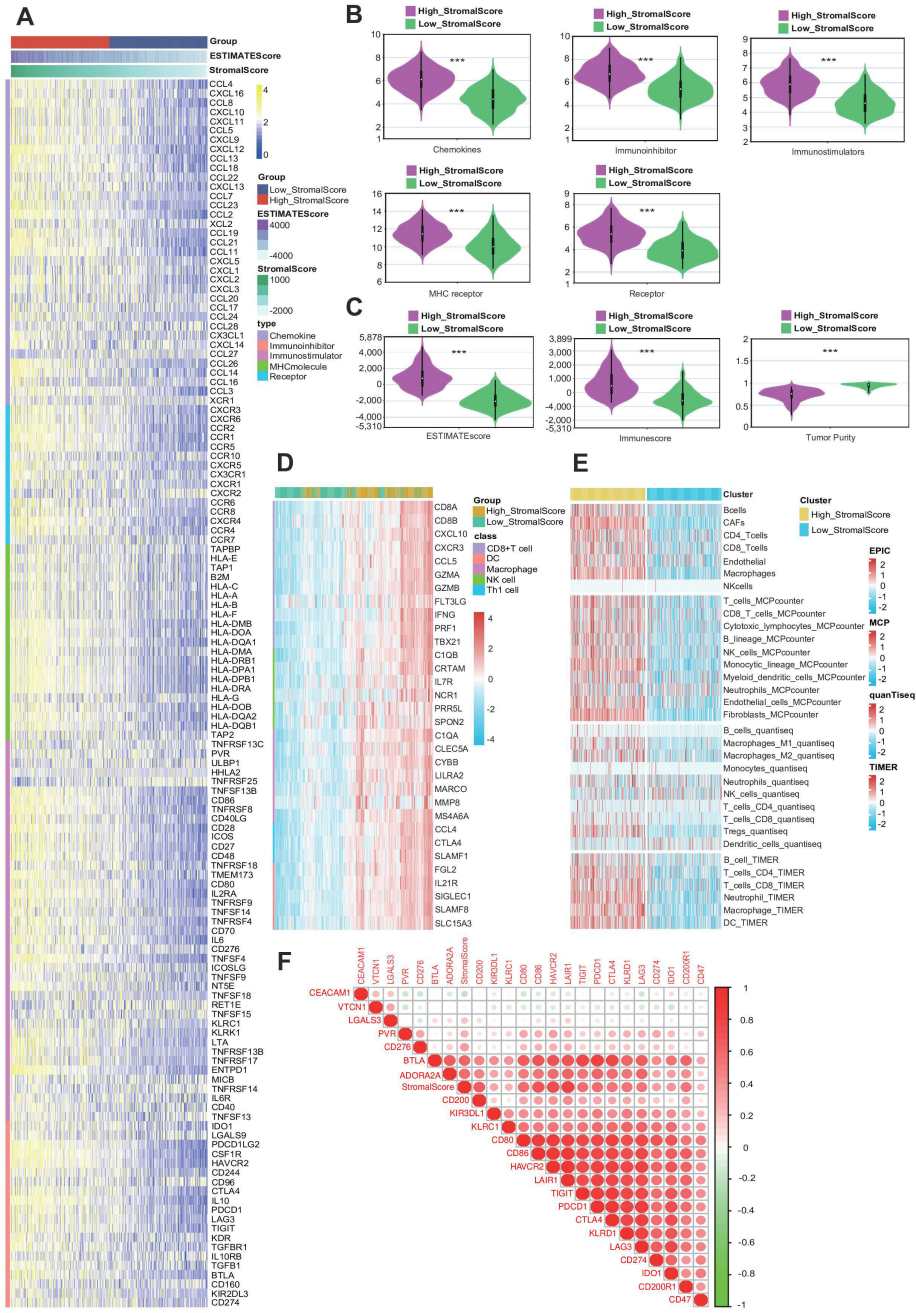
A high stroma is closely linked with an immunosuppressive tumor microenvironment (TME). **(A)** Heatmap plot of the expression levels of 133 immunomodulators in subgroups with different stromal scores in bladder cancer (BLCA). **(B)** Different violin plots of the expression of immunomodulators in subgroups with different stromal scores in BLCA. **(C)** Violin plots of the expression of the ESTIMATE score, immune score, and tumor purity of the Cancer Genome Atlas (TCGA)-BLCA dataset across subgroups with different stromal scores. **(D)** Heatmap plot of different gene markers of tumor-infiltrating immune cells (TIICs) in subgroups with different stromal scores. **(E)** Heatmap plot of TIICs calculated by four immune algorithms in subgroups with different stromal scores. **(F)** Triangle heatmap plot of the correlation between the stromal score and different immune checkpoints.

Given the high potential correlation between the stroma and several immune factors in BLCA (42), we investigated the relationship between stromal score and 133 immunomodulators, including chemokines, immunoinhibitors, immunostimulators, MHCs, and receptors. We found that the expression levels of immunomodulators were upregulated in the high-stromal score group, suggesting an important role of a high stroma in shaping the immunosuppressive TME (**Figure 3A**) (15). Next, the violin plots showed that the expression levels of immunomodulators were upregulated in the high-stromal score group (**Figure 3B**). Since the stromal score was calculated by the R package “ESTIMATE,” we also made a violin plot comparing the stromal score and other scores from the R package “ESTIMATE,” where we found out that the high-stromal score group exhibited a higher ESTIMATE score and immune score, accompanied by lower tumor purity (**Figure 3C**) (5). This indicates that tumors with a higher stroma are surrounded by more immune cells. A heatmap was generated to visualize the relationship between stromal score and gene markers of immune cells. The result indicates that tumors with a higher stroma tend to be infiltrated by more immune cells (**Figure 3D**). To further validate these findings, we applied several algorithms to estimate the infiltration levels of TIICs. The results show that the majority of TIIC infiltration levels were highly elevated in the samples in the high-stromal score group (**Figure 3E**). The triangle heatmap plot showed a high correlation between stromal score and common inhibitory immune checkpoints, including CD274, KLRC1, LAIR1, and CD47 (**Figure 3F**) (43). This result shows that the stromal score is closely correlated with the immune checkpoints. Overall, a high stroma was found to shape the immunosuppressive TME.

### The stroma predicts the immune phenotype in BLCA

**Figure 4:**
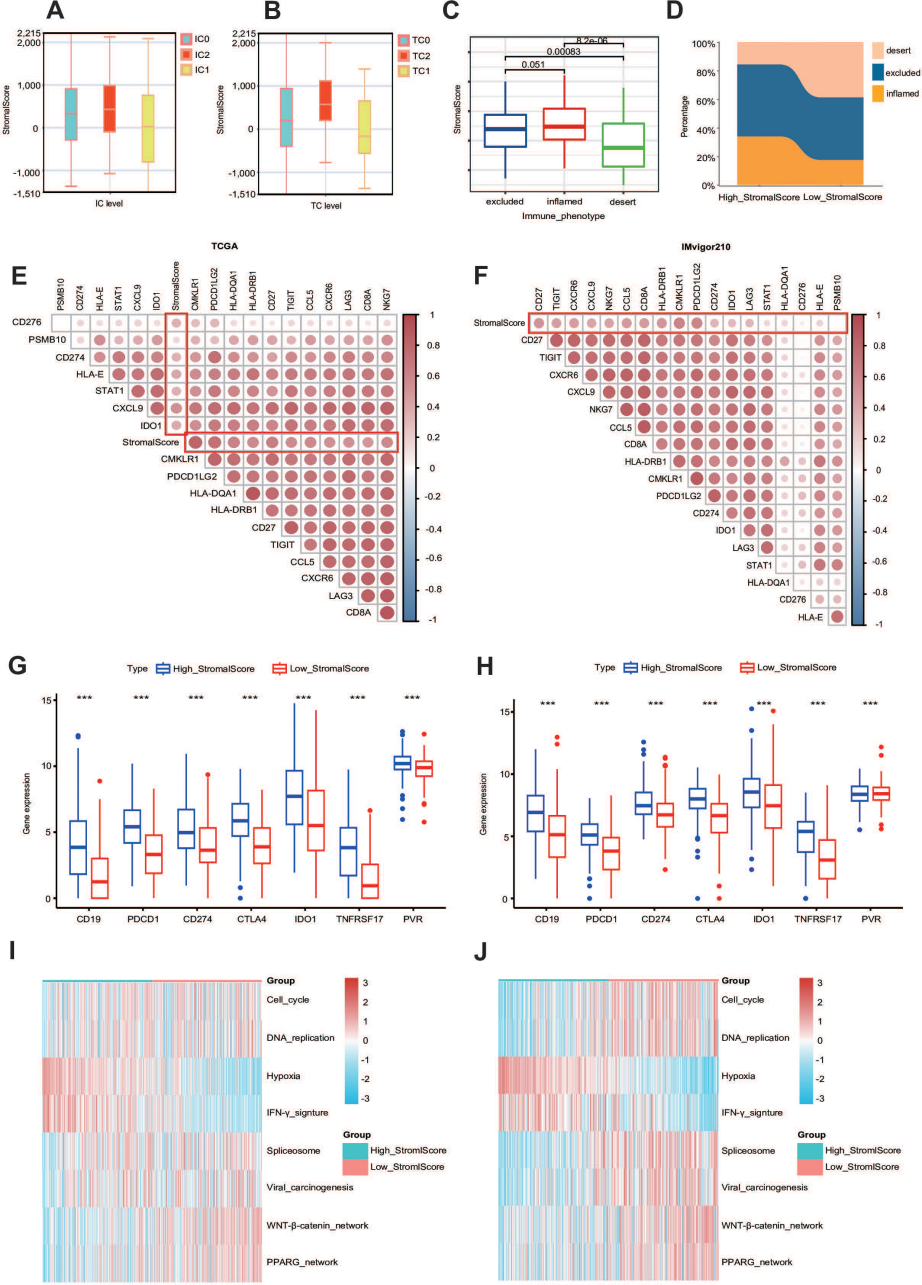
The stroma can predict the immune phenotype. **(A, B)** Stromal score expression in tumor cells (TCs) and immune cells (ICs) between subgroups with different stromal scores in the IMvigor210 dataset. **(C)** Expression of the stromal score in different immune phenotypes (excluded, inflamed, and desert phenotypes) in the IMvigor210 dataset. **(D)** Immunophenotypic distribution stratified by stromal scoring strata within the IMvigor210 immunotherapy cohort. **(E, F)** Triangle heatmap plot between T-cell-inflamed score genes and stromal score in the TCGA and IMvigor210 datasets. **(G, H)** The expression levels of different common immune targets in different stromalscore subgroups in TCGA-BLCA and IMvigor210 datasets. ***P ≤ 0.001. **(I, J)** Enrichment scores calculated by the “GSVA” R package of several immune signatures in subgroups with different stromal scores.

Given that a higher stroma is associated with higher expression of PD-L1, we hypothesized that patients with a higher stroma would have a better response to immunotherapy. We quoted the IMvigor210 cohort, which provided abundant data on PD-L1 expression in immune cells (ICs) and tumor cells (TCs), immunotypes of tumors, and response to immunotherapy, to explore the relationship between stromal score and immunotherapy (44). The results show that the stromal score was higher in the IC2 group (immune cells with the highest PD-L1 expression) and the TC2 group (tumor cells with the highest PD-L1 expression) (**Figures 4A, B**). We also analyzed the stromal score in different tumor immunotypes (excluding immunotypes, desert immunotypes, and inflamed immunotypes) and found that the stromal score was higher in the excluded group (**Figure 4C**) (45).

Additionally, we calculated the percentage of these three immunotypes and found that the excluded immunotype was the most prevalent in both the high- and low-stromal score groups. Importantly, the percentage of the excluded immunotype was higher in the high-stromal score group, which also validated our previous conclusion (**Figure 4D**). Since the stromal score was higher in the IC2 and TC2 groups, in order to better study the relationship between the two, we introduced the IMvigor210 cohort. We also identified a positive correlation between the stromal score and the T-cell-inflamed score gene list in both the TCGA cohort and the IMvigor210 cohort, including CD27, CD276, and CD274 (**Figures 4E, F**) (46). Heatmap analysis revealed significant associations between stromal indices and the candidate gene cluster. Notably, clinically validated immunotherapeutic targets including CD19 (B-cell marker), PDCD1 (PD-1), and CD274 (PD-L1) demonstrated consistently elevated expression patterns in the high-stromal score subgroups across both the TCGA and IMvigor210 cohorts (**Figures 4G, H**). More importantly, the stromal score demonstrated significant positive correlations with enrichment scores of the majority of immunotherapy-positive gene signatures, suggesting potential predictive value for treatment response stratification (**Figures 4I, J**). Thus, we demonstrated that the stromal score can effectively predict the immune phenotype in BLCA.

### The stroma predicts the molecular subtype and response to therapeutic choices in BLCA

**Figure 5:**
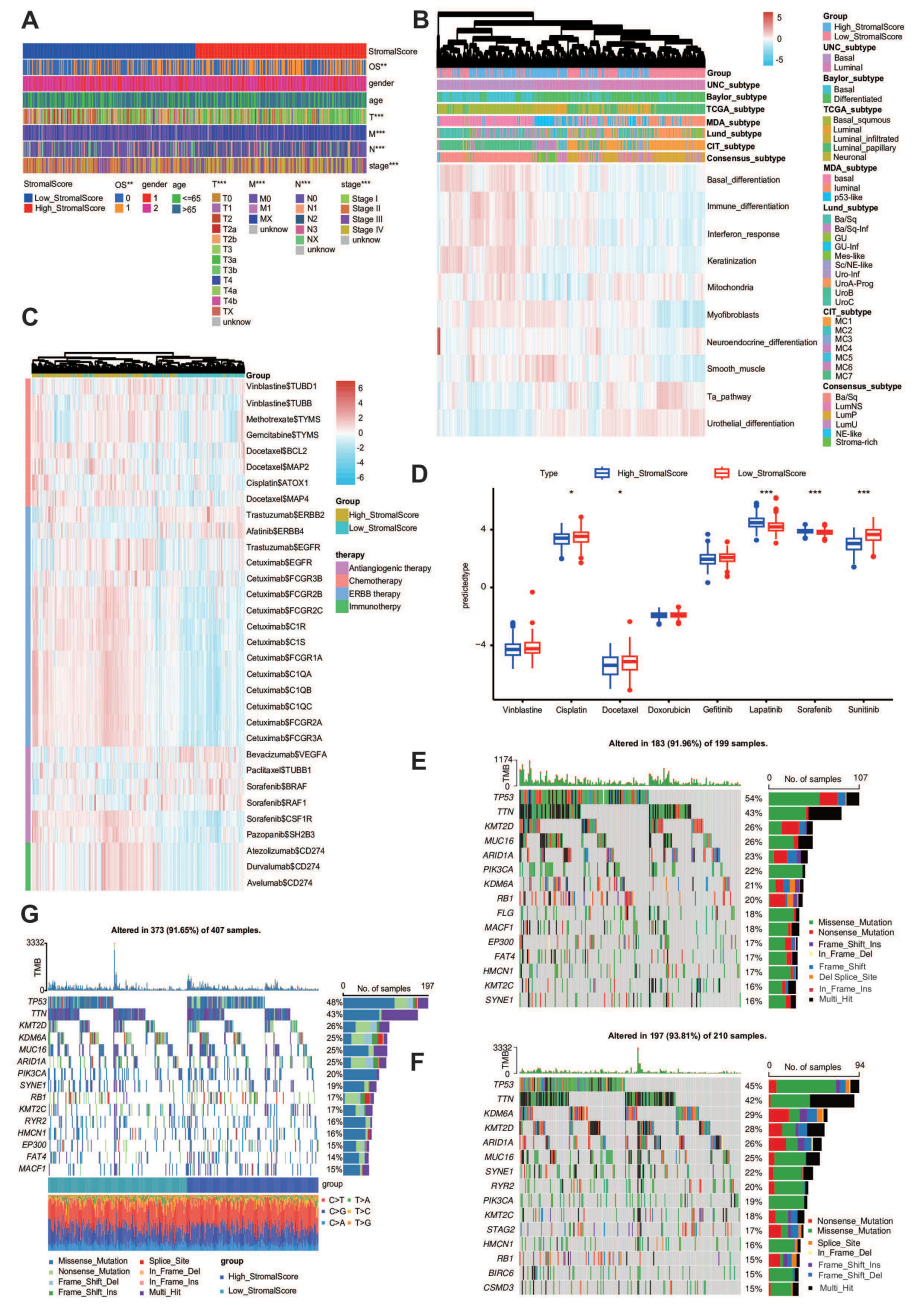
The stroma predicts the molecular subtype and response to therapeutic choices in BLCA. **(A)** The clinical heatmap plot between the stromal score and different clinicopathological features in BLCA. **(B)** The heatmap plot between the stromal score and molecular subtypes. **(C)** The heatmap plot between the stromal score and several drug-target genes. **(D)** The IC50 of common anticancer drugs in subgroups with different stromal scores. **(E, F)** Mutational profiles of chemotherapy-related genes in subgroups with different stromal scores in the TCGA-BLCA cohort.

Extensive genomic profiling research has established that basal-subclassified BLCA exhibits two distinct therapy advantages, namely, maximized intratumoral lymphocyte infiltration and superior objective response rates to PD-1 inhibitor therapies, including pembrolizumab (47). Notably, molecular taxonomy consensus frameworks further validate the enhanced clinical sensitivity of this molecular subtype to immune checkpoint inhibition strategies (48). As a result, we want to know whether the stroma could predict the molecular subtypes in BLCA. First, we evaluated the stroma and the clinicopathological features of BLCA (**Figure 5A**). The reason why we need to establish molecular subtypes is that they can predict the clinical response to chemotherapy, immunotherapy, and other therapies (14). According to the result shown in **Figure 5B**, we found that the stromal score was closely related to these subtypes, including the UNC, Consensus, CIT, Lund, MDA, Baylor, and TCGA subtypes (**Figure 5B**). To complement these findings, we systematically employed 10 computational frameworks alongside bladder cancer-specific molecular signatures to delineate stromal heterogeneity across stratification groups. In addition, we also made a heatmap correlation between the stromal score and the response to other therapies. The results from the DrugBank database (https://go.drugbank.com/) demonstrated enhanced efficacy of chemotherapeutic agents, EGFR-targeted therapies, and immunomodulators in the high-stromal score cohorts, whereas ERBB2/4 inhibitors and angiogenesis-blocking regimens exhibited diminished clinical benefit in this population (**Figure 5C**).

Utilizing the pRRophetic algorithm for pharmacogenomic prediction, we conducted systematic profiling of chemotherapeutic response disparities between stromal score-stratified cohorts. Heatmap analysis revealed that patients in the high-stromal score group demonstrated significantly enhanced therapeutic sensitivity in IC50 across multiple anticancer drugs, including cetuximab and vinblastine. These differential response patterns suggest that stromal score profiling may serve as a predictive biomarker for precision chemotherapy regimens (**Figure 5D**). Molecular classification systems demonstrated predictive capacity for multimodal therapeutic outcomes, particularly in neoadjuvant settings, encompassing cytotoxic chemotherapy, radiation protocols, and molecular-targeted interventions. Mechanistic analyses revealed that basal-phenotype malignancies exhibit biological predisposition to NAC responsiveness. Basal subtype tumors were more likely to respond to neoadjuvant chemotherapy (48, 49). We found that the mutation rates of TP53 and RB1 were significantly higher in the high-stromal score group (**Figure 5E**) and lower in the low-stromal score group (**Figure 5F**). The whole heatmap also showed that TP53 and RB1 were significantly higher (**Figure 5G**). Overall, the stroma is a novel classifier for the subtype of BLCA, meaning that patients with a high stroma tend to be sensitive to more therapy options.

### A high stroma microenvironment may lead to TME-immune overdrive

Logically, a high stroma TME was correlated with a worse prognosis, which was proven in our previous research. However, we also found that the stromal score was higher in the IC2 and TC2 groups, which is beneficial in the prognosis. We first made the violin plot to describe the tumor purity in both the TCGA-BLCA and IMvigor210 cohorts (50). The results show that the high-stromal score group showed lower tumor purity (**Supplementary Figure S2B**, **Figure 3C**). Why did the high-stromal score group show worse clinical outcome despite high immune cell infiltration and low tumor purity? To explain the logic between these two, we speculated that it may be related to immune overdrive (51). We focused on profiling IC gene expression patterns, based on the rationale that neoplastic cells exploit inhibitory checkpoint signaling pathways to attenuate T-cell responses and consequently shape an immunosuppressive TME (52). Notably, the high-stromal score group had the highest expression levels of ICs in both the TCGA-BLCA and IMvigor210 datasets (**Supplementary Figures S2C, D**). In addition, we found that the high-stromal score group showed higher TGF-β signaling in both the TCGA-BLCA and IMvigor210 cohorts (**Supplementary Figures S2E, F**), which has been confirmed as one of the mechanisms of immune evasion in previous studies (53). Analytical results demonstrated that patients with elevated stromal scores exhibited distinct TME characteristics marked by immunological hyperactivation. This phenotype is manifested through three hallmark features: pronounced leukocyte infiltration, diminished neoplastic cellularity, and elevated immune checkpoint expression. Our findings collectively suggest that stromal-enriched microenvironments may drive the TME toward an immune-overstimulated state, ultimately fostering a unique ecological niche characterized by lymphocyte abundance, tumor suppression, and upregulated expression of both immunoregulatory molecules and TGF-β.

### A high stroma microenvironment makes the tumor cells more proliferative, with stronger migration and invasion

**Figure 6:**
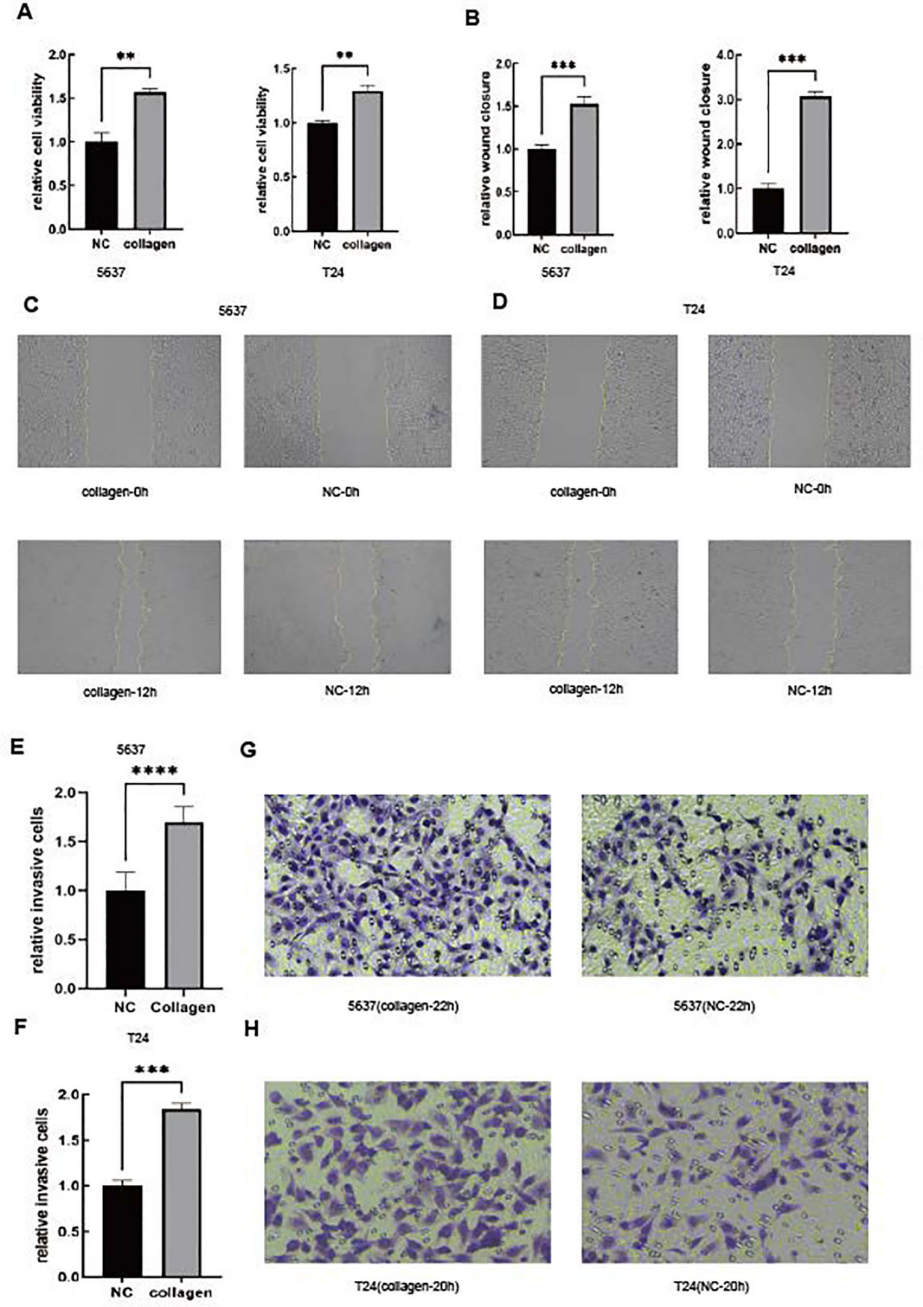
A high stroma microenvironment promotes BLCA cells to be more proliferative, migratory and invasive. **(A)** The cell viability of 5637 and T24 cells was determined by the CCK-8 assay. **(B–D)** The cell migration of 5637 and T24 cells was determined by the wound healing assay. **(E–H)** The cell invasion of 5637 and T24 cells was determined by the Boyden chamber assay.

As we all know, collagen is one of the most important components of the tumor microenvironment. Therefore, we used collagen to imitate the high-STR group (54, 55). In order to validate our conclusion, we performed the CCK-8 assay (**Figure 6A**). Furthermore, the CCK-8 results showed that the tumor’s relative cell viability in the collagen group was higher than in the NC group in both the 5637 and T24 cell lines. We also conducted the wound healing assay (**Figures 6B–D**) and the invasion assay (**Figures 6E–H**) in both the 5637 and T24 cell lines, illustrating that in the high-STR group, tumor cells tended to show higher migration and invasion, thus confirming our previous result. Both the 5637 and T24 cells showed in 12 h that the collagen group cells migrated more than the NC group cells in the wound healing assay. Moreover, 5637 (22 h) and T24 cells (20 h) showed that the collagen group cells were more invasive than the NC group cells in the invasion assay. The reason why a richer tumor stroma showed a worse prognosis remains to be determined.

### A high stroma is associated with clinical phenotypes in the in-house cohort

**Figure 7:**
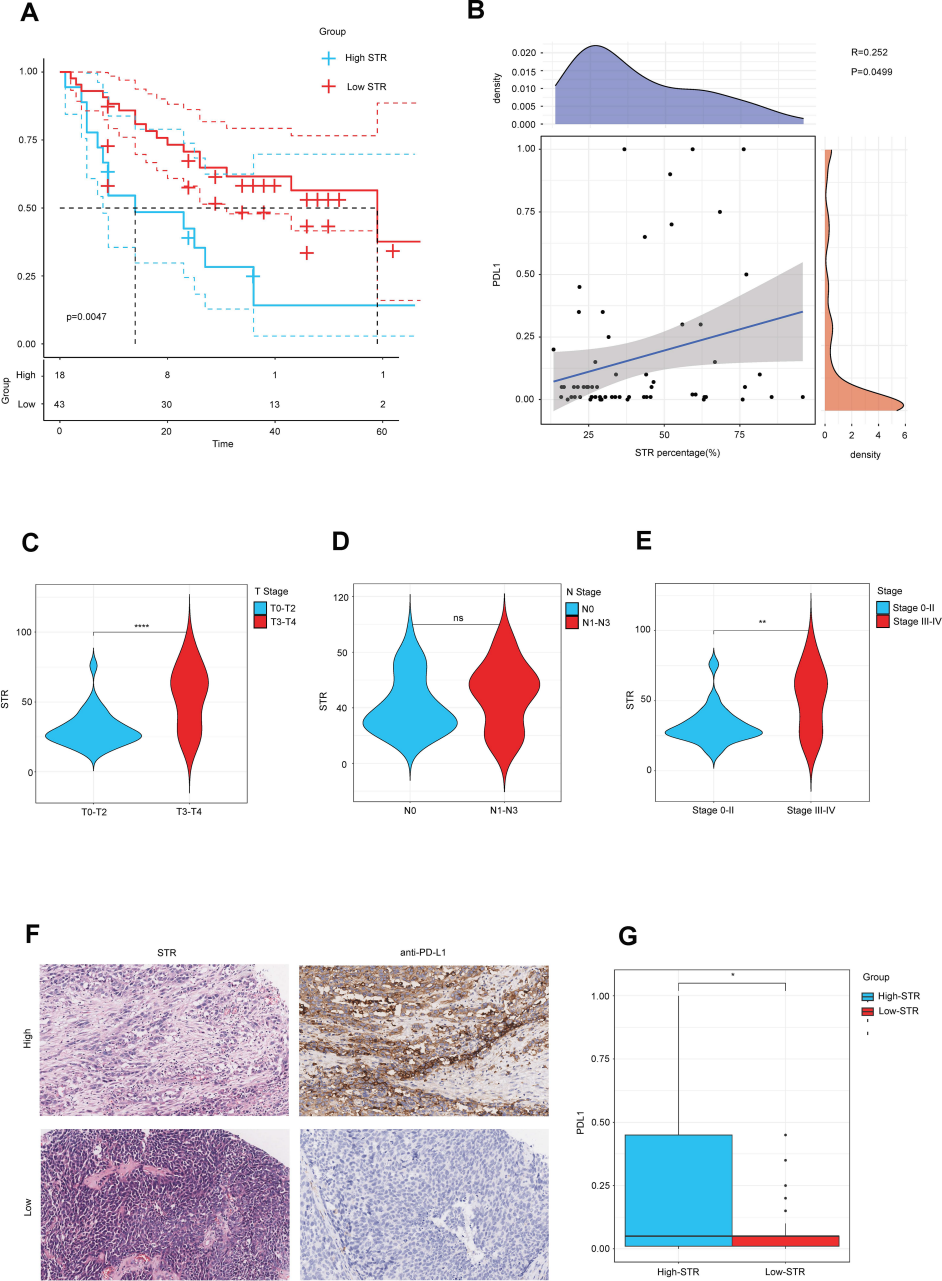
A higher stroma–tumor ratio (STR) is associated with clinical phenotypes in the in-house cohort. **(A)** The OS Kaplan–Meier analysis based on the optimal cutoff value between different STR subgroups. **(B)** Correlation plot between STR and PD-L1 expression in the in-house cohort. **(C–E)** STR in different subtypes such as T stages, N stages, and AJCC stages. **(F)** Immunohistochemical result of PD-L1 expression between different STR subgroups. **(G)** PD-L1 expression between different STR subgroups. ns, not significant.

In our previous study, we found that high STR was associated with high PD-L1. In order to verify our previous conclusion, we also obtained an in-house cohort to validate our conclusion, which included 61 BLCA samples. The Kaplan–Meier OS analysis based on the optimal cutoff value showed that the low-STR group survived longer than the high-STR group (**Figure 7A**). Furthermore, we calculated all the samples’ PD-L1 expression and real STR. The correlation plot between PD-L1 expression and STR showed that they were related (**Figure 7B**). Moreover, the violin plot of different subtypes showed that a higher grade, such as T3–T4 stage (**Figure 7C**), N1 stage (**Figure 7D**), and stage III–IV (**Figure 7E**), tended to have a higher STR. In addition, the current BLCA cohort was classified into low- and high-STR expression groups based on the median level of STR expression. The immunohistochemistry (IHC) results showed that the infiltrating level of PD-L1 was higher in the high-STR group (**Figure 7F**) and that PD-L1 expression was higher in the high-STR group (**Figure 7G**). In conclusion, a high stroma is closely associated with PD-L1expression levels and predicts the clinical phenotypes.

## Discussion

Existing research indicates that neoplastic tissues constitute architecturally complex ecosystems composed of malignant cells and diverse stromal constituents (56). Within solid malignancies, these supportive stromal elements engage in bidirectional signaling with transformed cells, collectively modulating neoplastic proliferation and metastatic potential (57). Significantly, malignant populations demonstrate remarkable plasticity in remodeling adjacent ECM components, ultimately establishing self-reinforcing microenvironments that are conducive to tumor progression and therapy resistance (58). The TME represents a sophisticated biological network integrating malignant clones with stromal cell populations, migratory immune effectors, soluble mediators, and structural biomolecules. Tumor–host interactions demonstrate paradoxical interdependence, simultaneously fostering mutualistic coevolution and competitive selection pressures. Crucially, microenvironmental components exhibit regulatory capacity over fundamental oncogenic processes including neoplastic transformation, clonal expansion, metastatic dissemination, and the emergence of treatment resistance (59). As one of the most important components of the TME, the tumor stroma plays a critical role in the occurrence and progression of tumors and treatment resistance, impacting numerous characteristics of cancer. These elements significantly influence antitumor immunity and are pivotal in determining the trajectory of tumor progression (60).

In this article, we studied the prognosis and immunity of BLCA in high- and low-stroma samples. We evaluated the richness of the stroma by the stromal score at the bioinformatics level and the tumor–stroma ratio at the pathological level. As shown by the majority of the studies, a higher stroma was usually linked with worse tumor prognosis and tumor invasiveness, which is associated with tumor immune escape in multiple cancers, such as female breast cancer (BCa) (61), hepatocellular carcinoma (62), and glioblastoma (63). A high stroma is closely linked with an immunosuppressive TME, which was validated by a heatmap showing the correlation among immunomodulators and several violin plots. Our comparative analysis of stromal-enriched specimens revealed a marked elevation in T-lymphocyte exhaustion signatures, with all eight evaluated phenotypic metrics demonstrating significantly higher values in the stromal-rich cohort. These patterns were particularly pronounced in progenitor-exhausted and terminally exhausted T-cell subsets. Mechanistic investigations have further confirmed the predominant exhausted status of tumor-infiltrating lymphocytes within stromal-dense microenvironments, correlating with progressive functional impairment. We found that the stroma could highly predict the molecular subtypes and therapy options in BLCA. In addition, we found that the stroma could predict the immune phenotype in BLCA, which was validated in the IMvigor210 cohort. From the above discussion, it can be concluded that STR could be an effective biomarker to predict the migration and invasion ability of tumor cells in BLCA.

In this study, we first used both the stromal score and STR to evaluate the richness of the tumor stroma, thus highlighting the value of STR in predicting the migration and invasion ability of tumor cells in BLCA. Furthermore, we also validated our bioinformatics results through the proliferation assay, wound healing assay, and invasion assay, showing that a high stroma may promote the migration and invasion of BLCA tumor cells. Moreover, we first associated a high stroma with the expression of PD-L1, which indicated that the tumor–stroma ratio can be a potential biomarker to identify tumors sensitive to immunotherapy. All of our results were also validated in our in-house cohort, showing that high stroma tumors were associated with a worse prognosis.

This result aligns with previous findings by Liu et al., who also observed that a high stroma may lead to a worse prognosis in tumors (17). Our study expands on their conclusion by demonstrating that high stroma tumors may predict a worse prognosis, thus predicting higher migration and invasion abilities of tumor cells. However, the relationship between the stroma and tumor immune escape has not been studied in BLCA. Moreover, we only validated our conclusion in BLCA instead of studying other kinds of tumors. We did not explore whether the migration and invasion abilities of cells will change under other collagen concentrations. This investigation acknowledges the methodological constraints arising from the restricted cohort size, potentially compromising the statistical power and external validity of observed associations. In addition, our in-house cohort’s samples were only enrolled from one hospital. As a result, there might still be shortcomings in the representativeness and generalizability of our in-house cohort. To overcome this limitation, we plan to expand our sample size by conducting a multicenter study. We also plan to combine STR with several common prognosis biomarkers of BLCA, such as TNM stage, to design a powerful and sensitive prognostic model. To achieve our model’s interpretive power and predictive capabilities, we intend to use machine learning to calculate and build our prognostic model, which will be the focus of our future work. The monocentric design further constrains the extrapolation of findings to populations with divergent demographic or clinicopathological characteristics. To enhance translational relevance, prospective multicenter investigations incorporating geographically diverse and demographically heterogeneous cohorts are warranted to corroborate these preliminary observations while ensuring methodological rigor.

Future research should explore the long-term effects of the stroma on tumor immunity and the TME in different populations to confirm our findings. Additionally, investigating the underlying mechanisms in more detail could provide a clearer understanding of how tumors impact BLCA migration and invasion. Understanding the functions of the stroma in tumor cells provides deeper insights into its potential as a biomarker.

## Conclusions

The current study reveals that a high stroma shapes a more immunosuppressive tumor microenvironment in BLCA and can predict different immune and clinical phenotypes in BLCA. Furthermore, a high stroma often predicted a higher stroma-to-parenchyma ratio, indicating a worse prognosis. Moreover, this study suggests that STR is a useful biomarker in predicting different molecular subtypes and therapy options in BLCA. Overall, we identify STR as a novel BLCA target for identifying tumor immunogenicity and providing better guidance on immunotherapy.

## Data Availability

The original contributions presented in the study are included in the article/**Supplementary Material**. Further inquiries can be directed to the corresponding authors.

## Supplementary material

Supplementary Figure 1 Pathological section of BLCA classified according to the stroma–tumor ratio. Samples with high stromal score scored as 1, such as TCGA-DK-A3IQ sample **(A)**, TCGA-FD-A5BS sample **(B)**, TCGA-XF-AAME sample **(C)** and TCGA-FD-A5BT sample **(D)**. Samples with low stromal score scored as 0, such as TCGA-ZF-AA4X sample **(E)**, TCGA-ZF-A9RM sample **(F)**, TCGA-E7-A5KF sample **(G)** and TCGA-ZF-AA4U sample **(H)**.

Supplementary Figure 2 High stroma microenvironment may lead to TME immune overdrive. **(A)** The t-test result between different STR subgroups. **(B)** Violin plot of tumor purity between subgroups with different stromal scores. **(C, D)** Expression levels of ICs of subgroups with different stromal scores in both TCGA-BLCA dataset and IMvigor210 dataset. **(E, F)** Expression of TGF-β signaling in both TCGA and IMvigor210 dataset.

## References

[1] Chen YT Tu WJ Ye ZH Wu CC Ueng SH Yu KJ. Integration of the cancer cell secretome and transcriptome reveals potential noninvasive diagnostic markers for bladder cancer. Proteomics Clin Appl. (2024) 18. doi: 10.1002/prca.202300033, PMID: 38196148

[2] Xia C Dong X Li H Cao M Sun D He S. Cancer statistics in China and United States, 2022: profiles, trends, and determinants. Chin Med J. (2022) 135:584–90. doi: 10.1097/CM9.0000000000002108, PMID: 35143424 PMC8920425

[3] Zhang Q Qi T Long Y Li X Yao Y Wu Q. GATA3 predicts the tumor microenvironment phenotypes and molecular subtypes for bladder carcinoma. Front Surg. (2022) 9. doi: 10.3389/fsurg.2022.860663, PMID: 35647011 PMC9135132

[4] Bhattacharjee S Hamberger F Ravichandra A Miller M Nair A Affo S. Tumor restriction by type I collagen opposes tumor- promoting effects of cancer-associated fibroblasts. J Clin Invest. (2021) 131. doi: 10.1172/JCI146987, PMID: 33905375 PMC8159701

[5] Zhang H Liu Y Xu Z Chen Q. miR-873 and miR-105–2 may affect the tumour microenvironment and are potential biomarkers for lung adenocarcinoma. Int J Gen Med. (2022) 15:3433–45. doi: 10.2147/IJGM.S352120, PMID: 35378915 PMC8976495

[6] Yang L Chen P Zhang L Wang L Sun T Zhou L. Prognostic value of nucleotyping, DNA ploidy and stroma in high-risk stage II colon cancer. Br J Cancer. (2020) 123:973–81. doi: 10.1038/s41416-020-0974-8, PMID: 32624576 PMC7492254

[7] Gutman DA Khalilia M Lee S Nalisnik M Mullen Z Beezley J. The digital slide archive: A software platform for management, integration, and analysis of histology for cancer research. Cancer Res. (2017) 77:E75–8. doi: 10.1158/0008-5472.CAN-17-0629, PMID: 29092945 PMC5898232

[8] Yoshihara K Shahmoradgoli M Martínez E Vegesna R Kim H Torres-Garcia W. Inferring tumour purity and stromal and immune cell admixture from expression data. Nat Commun. (2013) 4. doi: 10.1038/ncomms3612, PMID: 24113773 PMC3826632

[9] Jian Y Chen Q Al-Danakh A Xu Z Xu C Sun X. Identification and validation of sialyltransferase ST3Gal5 in bladder cancer through bioinformatics and experimental analysis. Int Immunopharmacol. (2024) 138. doi: 10.1016/j.intimp.2024.112569, PMID: 38959540

[10] Wang J Xie Y Qin D Zhong S Hu X. CXCL12, a potential modulator of tumor immune microenvironment (TIME) of bladder cancer: From a comprehensive analysis of TCGA database. Front Oncol. (2022) 12. doi: 10.3389/fonc.2022.1031706, PMID: 36419891 PMC9676933

[11] Su H Karin M. Collagen architecture and signaling orchestrate cancer development. Trends Cancer. (2023) 9:764–73. doi: 10.1016/j.trecan.2023.06.002, PMID: 37400314

[12] Liu J Pan D Huang X Wang S Chen H Zhu YZ. Targeting collagen in tumor extracellular matrix as a novel targeted strategy in cancer immunotherapy. Front Oncol. (2023) 13. doi: 10.3389/fonc.2023.1225483, PMID: 37692860 PMC10484796

[13] Wherry EJ Kurachi M. Molecular and cellular insights into T cell exhaustion. Nat Rev Immunol. (2015) 15:486–99. doi: 10.1038/nri3862, PMID: 26205583 PMC4889009

[14] Hu J Yu A Othmane B Qiu D Li H Li C. Siglec15 shapes a non-inflamed tumor microenvironment and predicts the molecular subtype in bladder cancer. Theranostics. (2021) 11:3089–108. doi: 10.7150/thno.53649, PMID: 33537076 PMC7847675

[15] Mei J Cai Y Xu R Zhu Y Zhao X Zhang Y. Protocol to identify novel immunotherapy biomarkers based on transcriptomic data in human cancers. STAR Protoc. (2023) 4:102258–8. doi: 10.1016/j.xpro.2023.102258, PMID: 37119142 PMC10173013

[16] Mariathasan S Turley SJ Nickles D Castiglioni A Yuen K Wang Y. TGFβ attenuates tumour response to PD-L1 blockade by contributing to exclusion of T cells. Nature. (2018) 554:544–+. doi: 10.1038/nature25501, PMID: 29443960 PMC6028240

[17] Liu L Xu L Wu D Zhu Y Li X Xu C. Impact of tumour stroma-immune interactions on survival prognosis and response to neoadjuvant chemotherapy in bladder cancer. Ebiomedicine. (2024) 104. doi: 10.1016/j.ebiom.2024.105152, PMID: 38728838 PMC11090066

[18] Lv W Liu L Zhang W Chen L Ma D Chen N. Kaplan-Meier method based accident occurrence time interval calculating method, involves establishing sample matrix to process full sample matrix, and obtaining function estimator and risk function to determine driver survival time interval. Jiangsu Intelligent Transportation Syste.

[19] Shi Y Chen S Xing H Jiang G Wu N Liu Q. Comprehensive analysis of prognostic microenvironment-related genes in invasive breast cancer. Front Oncol. (2022) 11. doi: 10.3389/fonc.2021.576911, PMID: 35047378 PMC8761742

[20] Xu J Tang L Wang Z Zhang Q Jiang Y. MIR548P and TRAV39 are potential indicators of tumor microenvironment and novel prognostic biomarkers of esophageal squamous cell carcinoma. J Oncol. (2022) 2022. doi: 10.1155/2022/3152114, PMID: 36164348 PMC9509226

[21] Charoentong P Finotello F Angelova M Mayer C Efremova M Rieder D. Pan-cancer immunogenomic analyses reveal genotype-immunophenotype relationships and predictors of response to checkpoint blockade. Cell Rep. (2017) 18:248–62. doi: 10.1016/j.celrep.2016.12.019, PMID: 28052254

[22] Ayers M Lunceford J Nebozhyn M Murphy E Loboda A Kaufman DR. IFN-γ-related mRNA profile predicts clinical response to PD-1 blockade. J Clin Invest. (2017) 127:2930–40. doi: 10.1172/JCI91190, PMID: 28650338 PMC5531419

[23] Xu M Zhang T Xia R Wei Y Wei X Chen Q. TIMER2.0 for analysis of tumor-infiltrating immune cells. Nucleic Acids Res. (2020) 48:W509–14. doi: 10.1093/nar/gkaa407, PMID: 32442275 PMC7319575

[24] Racle J Gfeller D. EPIC: A tool to estimate the proportions of different cell types from bulk gene expression data. Methods Mol Biol (Clifton NJ). (2020) 2120:233–48. doi: 10.1007/978-1-0716-0327-7_17, PMID: 32124324

[25] Petitprez F Vano YA Becht E Giraldo NA de Reyniès A Sautès-Fridman C. Transcriptomic analysis of the tumor microenvironment to guide prognosis and immunotherapies. Cancer Immunol Immunother. (2018) 67:981–8. doi: 10.1007/s00262-017-2058-z, PMID: 28884365 PMC11028160

[26] Finotello F Mayer C Plattner C Laschober G Rieder D Hackl H. Molecular and pharmacological modulators of the tumor immune contexture revealed by deconvolution of RNA-seq data. Genome Med. (2019) 11:34–4. doi: 10.1186/s13073-019-0638-6, PMID: 31126321 PMC6534875

[27] Shi X Peng X Chen Y Shi Z Yue C Zuo L. Overexpression of MTHFD2 represents an inflamed tumor microenvironment and precisely predicts the molecular subtype and immunotherapy response of bladder cancer. Front Immunol. (2023) 14. doi: 10.3389/fimmu.2023.1326509, PMID: 38130721 PMC10733511

[28] Feng B Lin J Robinson M Sun WK Sun W. Identifying a human tumor as likely to be responsive or non-responsive to treatment with tivozanib by measuring, in a tissue sample, the relative expression level of genes in a hypoxia signature, and calculating a hypoxia signature. Aveo Pharm Inc.

[29] Ayers MD Cristrscu R Loboda A Lunceford JK Ma H Mcclanahan TK. Testing a tumor for the presence or absence of a biomarker that predicts response to treatment with a PD-1 antagonist, comprises obtaining a sample from the tumor, measuring the raw RNA expression level in the tumor sample. World Intellectual Property Organization Patent, WO2021091747-A1. (2021).

[30] Deng L Li S Huang L Luo H Hong Z Hu B. EMT simulation method for power system used for, such as system design, involves obtaining module execution requirement corresponding to each EMT sub-module, determining target hardware simulator corresponding to EMT sub-module. Csg Electric Power Res Inst Co Ltd (Cspg-C) China Southern Power Grid Co Ltd (Cspg-C.

[31] Cruz T Mendoza N Casas-Recasens S Noell G Hernandez-Gonzalez F Frino-Garcia A. Lung immune signatures define two groups of end-stage IPF patients. Respir Res. (2023) 24. doi: 10.1186/s12931-023-02546-8, PMID: 37770891 PMC10540496

[32] Li J Miao B Wang S Dong W Xu H Si C. Hiplot: a comprehensive and easy-to-use web service for boosting publication-ready biomedical data visualization. Briefings Bioinf. (2022) 23. doi: 10.1093/bib/bbac261, PMID: 35788820

[33] Ma RC Szurszewski JH. Cholecystokinin depolarizes neurons of cat pancreatic ganglion. Peptides. (1996) 17:775–83. doi: 10.1016/0196-9781(96)00078-2, PMID: 8844766

[34] Martinotti S Ranzato E. Scratch wound healing assay. Methods Mol Biol (Clifton NJ). (2020) 2109:225–9. doi: 10.1007/7651_2019_259, PMID: 31414347

[35] Marshall J. Transwell() invasion assays. Methods Mol Biol (Clifton NJ). (2011) 769:97–110. doi: 10.1007/978-1-61779-207-6_8, PMID: 21748672

[36] Wang R. Testing with p*-values: Between p-values, mid p-values, and e-values. Bernoulli. (2024) 30:1313–46. doi: 10.3150/23-BEJ1633

[37] Han B Zhang C Zhang X. Method for preparing bovine corneal stroma that is utilized in surgery, involves isolating cornea from bovine eye, followed by immersing in hypotonic solution, processing, rinsing, irradiating and subjecting to dry preservation. Beijing Saier Taihe Biomedical Technolog.

[38] Cao Y Hu X Wang M. Production method of transplanting material for implantation operation of living body corneal stroma ring, includes preparing biological support, culturing living body corneal stroma cell, and preparing corneal stroma cell support material. Univ No 9 People Hospital Attached to Sh; Univ Shanghai Second Medical.

[39] Plante J-F. About an adaptively weighted Kaplan-Meier estimate. Lifetime Data Anal. (2009) 15:295–315. doi: 10.1007/s10985-009-9120-x, PMID: 19533346

[40] Si J Shi X Sun S Zou B Li Y An D. Hematopoietic progenitor kinase1 (HPK1) mediates T cell dysfunction and is a druggable target for T cell-based immunotherapies. Cancer Cell. (2020) 38:551–+. doi: 10.1016/j.ccell.2020.08.001, PMID: 32860752

[41] Ni Y Jiang M Wu Y Xiao P Wu A Xia W. Anoikis-related CTNND1 is associated with immuno- suppressive tumor microenvironment and predicts unfavorable immunotherapeutic outcome in non-small cell lung cancer. J Cancer. (2024) 15:317–31. doi: 10.7150/jca.89542, PMID: 38169514 PMC10758022

[42] Jung M Rose M Knuechel R Loeffler C Muti H Kather JN. Characterisation of tumour-immune phenotypes and PD-L1 positivity in squamous bladder cancer. BMC Cancer. (2023) 23. doi: 10.1186/s12885-023-10576-0, PMID: 36726072 PMC9890720

[43] Ganjalikhani-Hakemi M Xu L Zamyatnin AA Bazhin AV. Editorial: cellular and molecular mechanisms of immune checkpoint blockers in anti-leukemia/lymphoma immune therapy. Front Oncol. (2022) 12. doi: 10.3389/fonc.2022.872300, PMID: 35321430 PMC8936063

[44] Pyo JS Ko SH Ko YS Kim NY. Clinicopathological significance of PD-L1 expression in colorectal cancer: Impact of PD-L1 expression on pFOXO1 expression. Pathol Res Pract. (2020) 216. doi: 10.1016/j.prp.2019.152764, PMID: 31836325

[45] Li TE Zhang Z Wang Y Xu D Dong J Zhu Y. A novel immunotype-based risk stratification model predicts postoperative prognosis and adjuvant TACE benefit in Chinese patients with hepatocellular carcinoma. J Cancer. (2021) 12:2866–76. doi: 10.7150/jca.54408, PMID: 33854587 PMC8040877

[46] Yang S-D Park H-S. TcellInflamedDetector: an R package to distinguish T cell inflamed tumor types from non-T cell inflamed tumor types. Genomics Inf. (2022) 20:e13–3. doi: 10.5808/gi.22005, PMID: 35399012 PMC9001994

[47] Necchi A Raggi D Gallina A Ross JS Farè E Giannatempo P. Impact of molecular subtyping and immune infiltration on pathological response and outcome following neoadjuvant pembrolizumab in muscle -invasive bladder cancer. Eur Urol. (2020) 77:701–10. doi: 10.1016/j.eururo.2020.02.028, PMID: 32165065

[48] Kamoun A de Reyniès A Allory Y Sjödahl G Robertson AG Seiler R. A consensus molecular classification of muscle-invasive bladder cancer. Eur Urol. (2020) 77:420–33. doi: 10.1016/j.eururo.2019.09.006, PMID: 31563503 PMC7690647

[49] McConkey DJ Choi W Shen Y Lee IL Porten S Matin SF. A prognostic gene expression signature in the molecular classification of chemotherapy-naive urothelial cancer is predictive of clinical outcomes from neoadjuvant chemotherapy: A phase 2 trial of dose-dense methotrexate, vinblastine, doxorubicin, and cisplatin with bevacizumab in urothelial cancer. Eur Urol. (2016) 69:855–62. doi: 10.1016/j.eururo.2015.08.034, PMID: 26343003 PMC4775435

[50] Qin Y Feng H Chen M Wu H Zheng X. InfiniumPurify: An R package for estimating and accounting for tumor purity in cancer methylation research. Genes Dis. (2018) 5:43–5. doi: 10.1016/j.gendis.2018.02.003, PMID: 30258934 PMC6147081

[51] Cui K Yao S Zhang H Zhou M Liu B Cao Y. Identification of an immune overdrive high-risk subpopulation with aberrant expression of FOXP3 and CTLA4 in colorectal cancer. Oncogene. (2021) 40:2130–45. doi: 10.1038/s41388-021-01677-w, PMID: 33627780

[52] Riera-Domingo C Audigé A Granja S Cheng WC Ho PC Baltazar F. Immunity, hypoxia, and metabolism-the menage a trois of cancer: implications for immunotherapy. Physiol Rev. (2020) 100:1–102. doi: 10.1152/physrev.00018.2019, PMID: 31414610

[53] Guinney J Dienstmann R Wang X de Reyniès A Schlicker A Soneson C. The consensus molecular subtypes of colorectal cancer. Nat Med. (2015) 21:1350–6. doi: 10.1038/nm.3967, PMID: 26457759 PMC4636487

[54] Han X Burke RM Zettel ML Tang P Brown EB. Second harmonic properties of tumor collagen: determining the structural relationship between reactive stroma and healthy stroma. Optics Express. (2008) 16:1846–59. doi: 10.1364/OE.16.001846, PMID: 18542263

[55] Baldari S Di Modugno F Nisticò P Toietta G. Strategies for efficient targeting of tumor collagen for cancer therapy. Cancers. (2022) 14. doi: 10.3390/cancers14194706, PMID: 36230627 PMC9563908

[56] Yang J. Tumor targeted molecule used for imaging contrast agent for pharmaceutical composition used for treating, e.g. liver cancer, comprises ferritin that can specifically target combined with tumor cell or tumor tissue.

[57] Kim K Kim KM Jin J. Method for predicting the tumor-stroma ratio of cancer tissue using deep learning model, involves calculating tumor-stroma ratio of target tissue based on first binary image and second binary image by analyzer. Samsung Life Public Welfare Found Med Ce.

[58] Xu M Zhang T Xia R Wei Y Wei X. Targeting the tumor stroma for cancer therapy. Mol Cancer. (2022) 21. doi: 10.1186/s12943-022-01670-1, PMID: 36324128 PMC9628074

[59] Shi X Wang X Yao W Shi D Shao X Lu Z. Mechanism insights and therapeutic intervention of tumor metastasis: latest developments and perspectives. Signal Transduct Target Ther. (2024) 9. doi: 10.1038/s41392-024-01885-2, PMID: 39090094 PMC11294630

[60] Huang G Li B. New substituted piperazine based tumor stroma imaging agents useful as fibroblast activation protein inhibitor in medicament for diagnosis and treatment of various tumor stroma and Malignant tumors. Univ Shanghai Medicine & Health Sci.

[61] Chen Y Meng Z Zhang L Liu F. CD2 is a novel immune-related prognostic biomarker of invasive breast carcinoma that modulates the tumor microenvironment. Front Immunol. (2021) 12. doi: 10.3389/fimmu.2021.664845, PMID: 33968066 PMC8102873

[62] Sun L Liu Z Wu Z Ning K Hu J Chen Z. Molecular subtype identification and signature construction based on Golgi apparatus-related genes for better prediction prognosis and immunotherapy response in hepatocellular carcinoma. Front Immunol. (2023) 14. doi: 10.3389/fimmu.2023.1113455, PMID: 37051238 PMC10083374

[63] Zhang H Bao M Liao D Zhang Z Tian Z Yang E. Identification of INSRR as an immune-related gene in the tumor microenvironment of glioblastoma by integrated bioinformatics analysis. Med Oncol. (2023) 40. doi: 10.1007/s12032-023-02023-8, PMID: 37099121

