# Supplementary material for: A high stroma-tumor ratio is associated with an immunosuppressive tumor microenvironment and a poor prognosis in bladder cancer
Source: Front Oncol. 2025 Aug 22;15:1604609. doi: 10.3389/fonc.2025.1604609 (PMC12411155; doi:10.3389/fonc.2025.1604609)
Supplement: Supplementary Figure 1 — Pathological section of BLCA classified according to the stroma–tumor ratio. Samples with high stromal score scored as 1, such as TCGA-DK-A3IQ sample (A), TCGA-FD-A5BS sample (B), TCGA-XF-AAME sample (C) and TCGA-FD-A5BT sample (D). Samples with low stromal score scored as 0, such as TCGA-ZF-AA4X sample (E), TCGA-ZF-A9RM sample (F), TCGA-E7-A5KF sample (G) and TCGA-ZF-AA4U sample (H). [file Image1.pdf]

High-STR Group

A

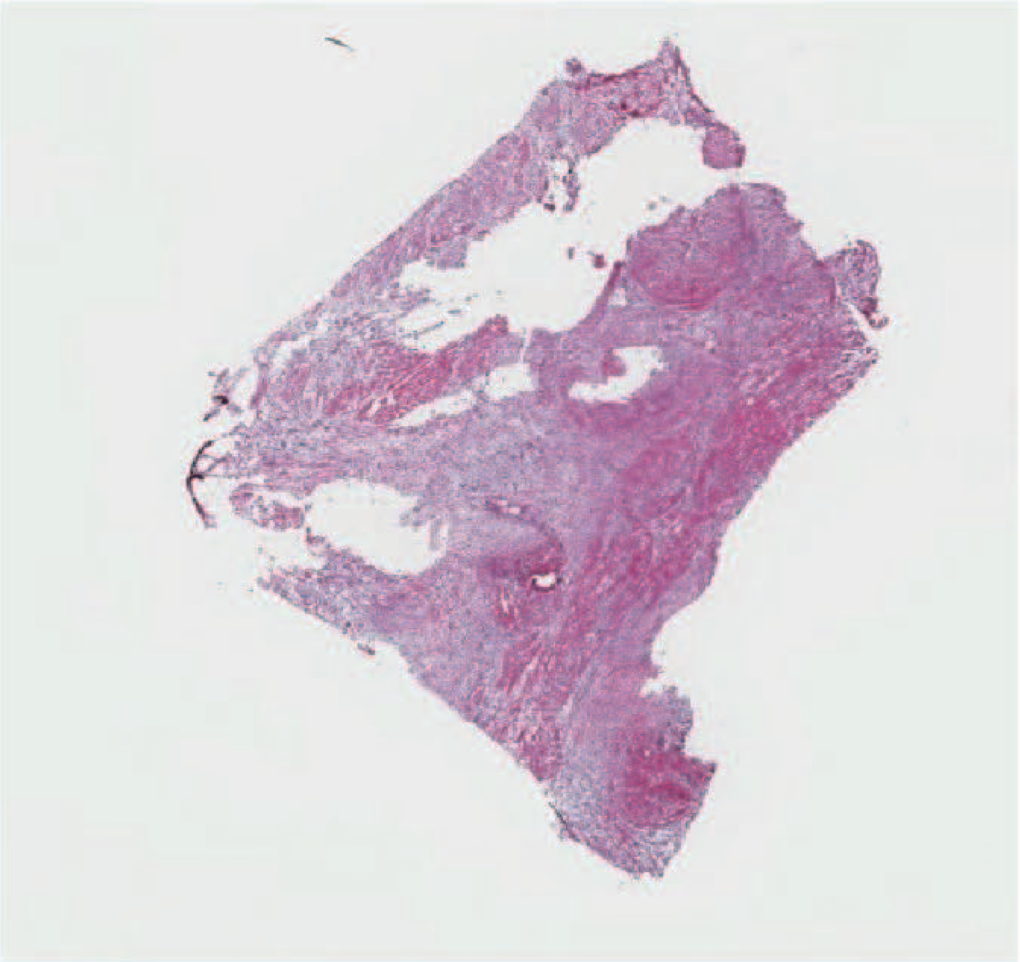

TCGA-DK-A3IQ Stromalscore： 1732.701621

B

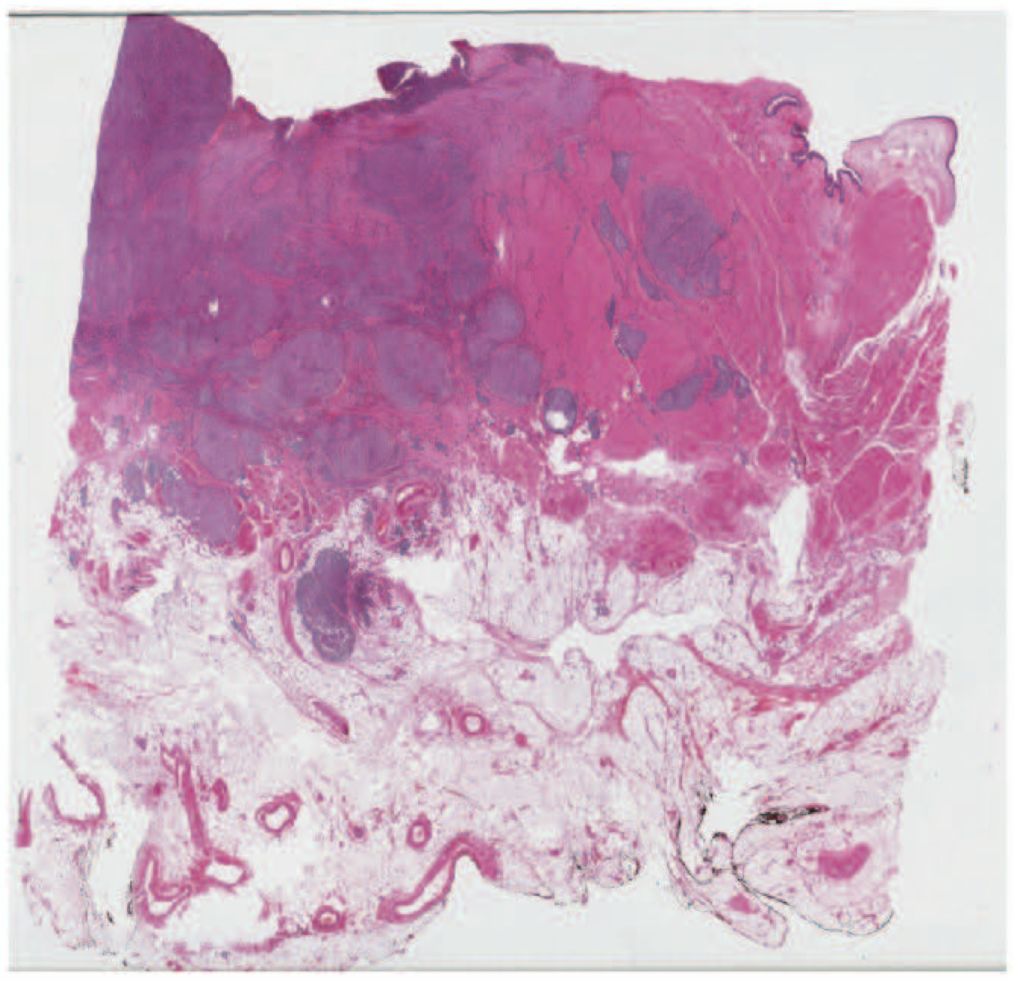

TCGA-FD-A5BS Stromalscore： 1619.510962

C

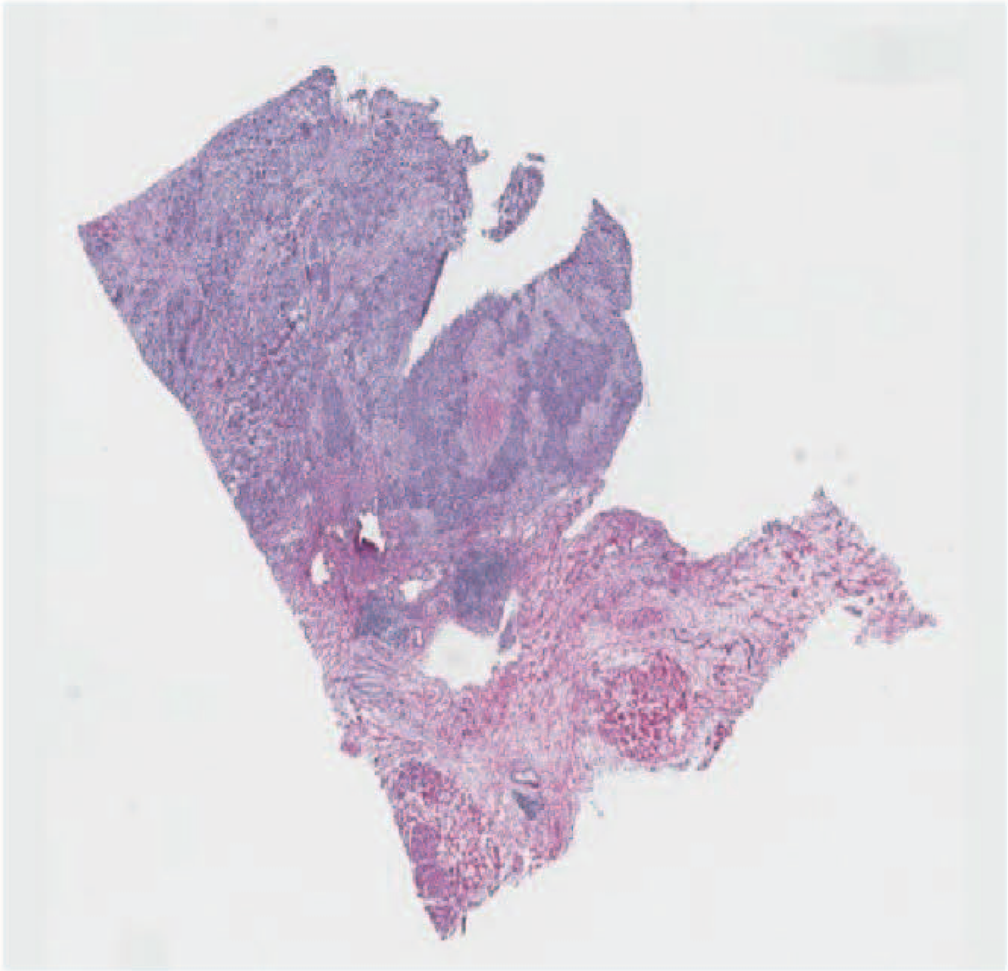

TCGA-XF-AAME Stromalscore： 1504.343847

D

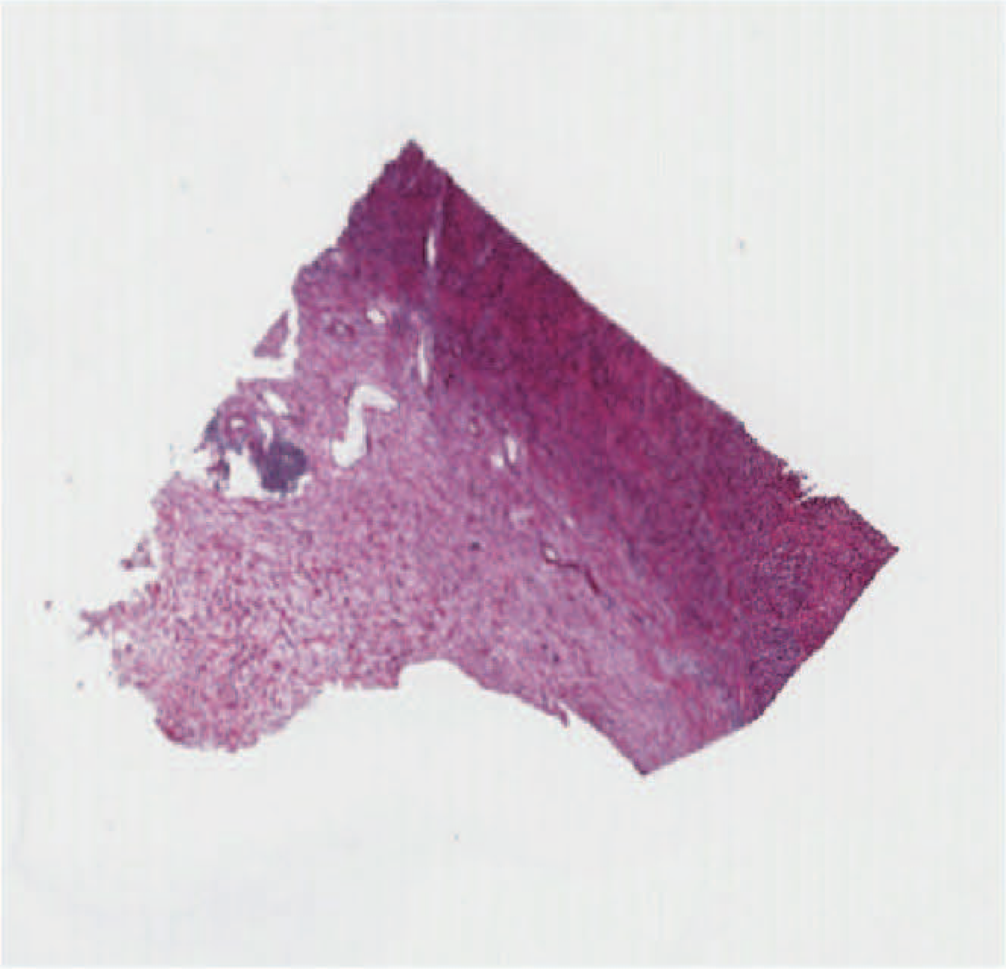

TCGA-FD-A5BT Stromalscore： 1459.421771

Low-STR Group

E

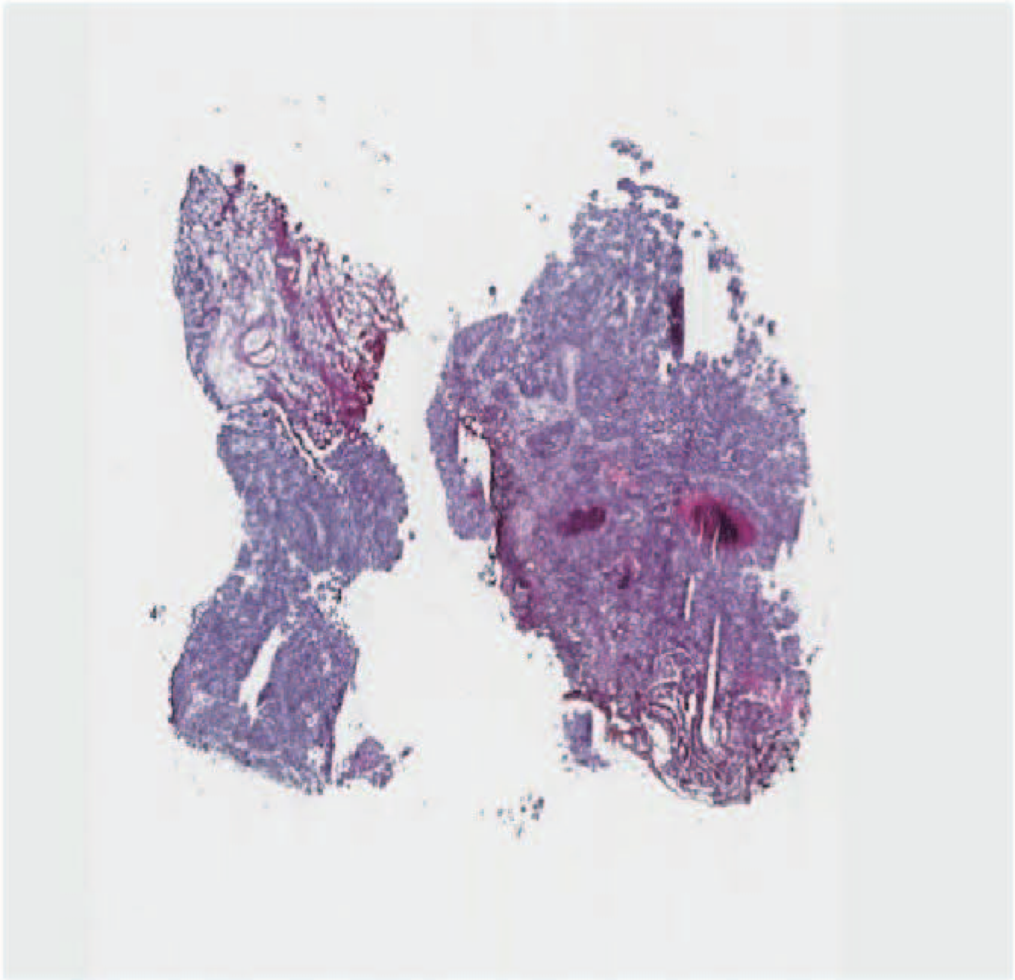

TCGA-ZF-AA4X Stromalscore： -2559.894485

F

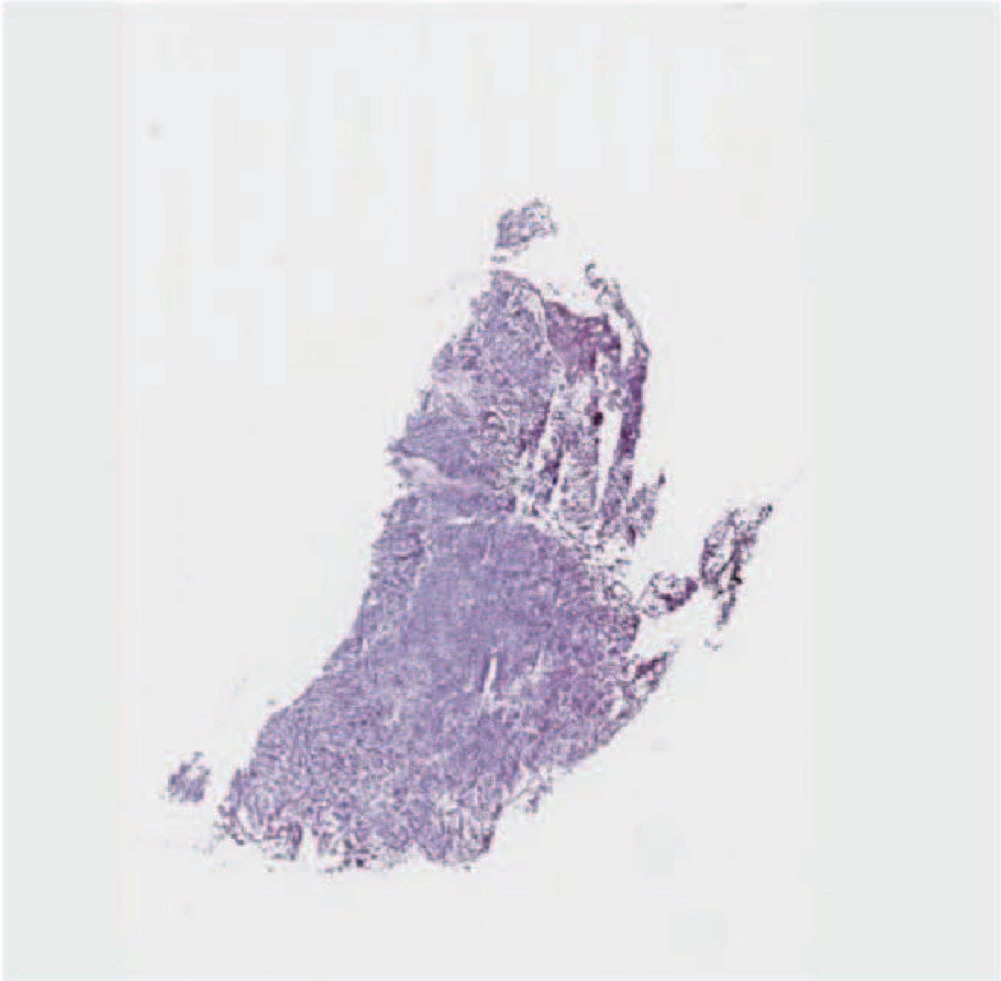

TCGA-ZF-A9RM Stromalscore： -2548.649232

G

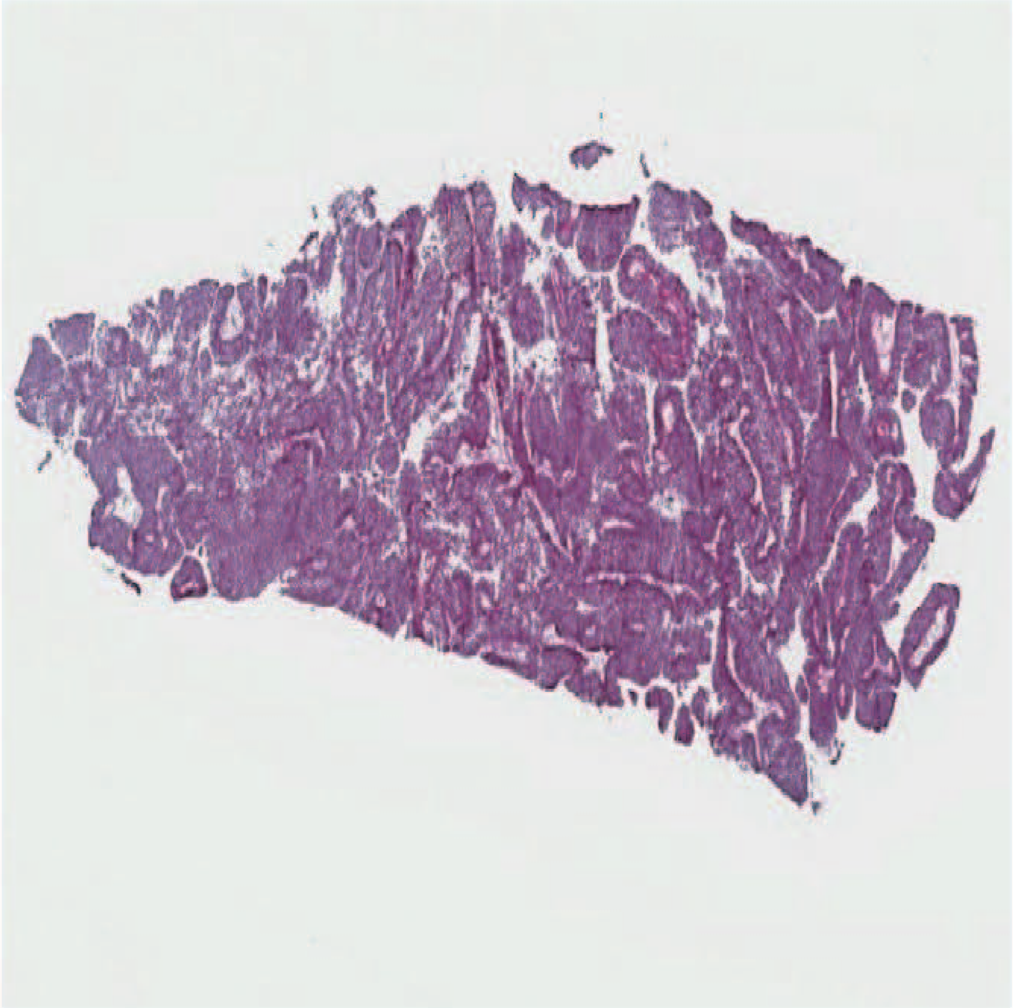

TCGA-E7-A5KF Stromalscore： -2469.531255

H

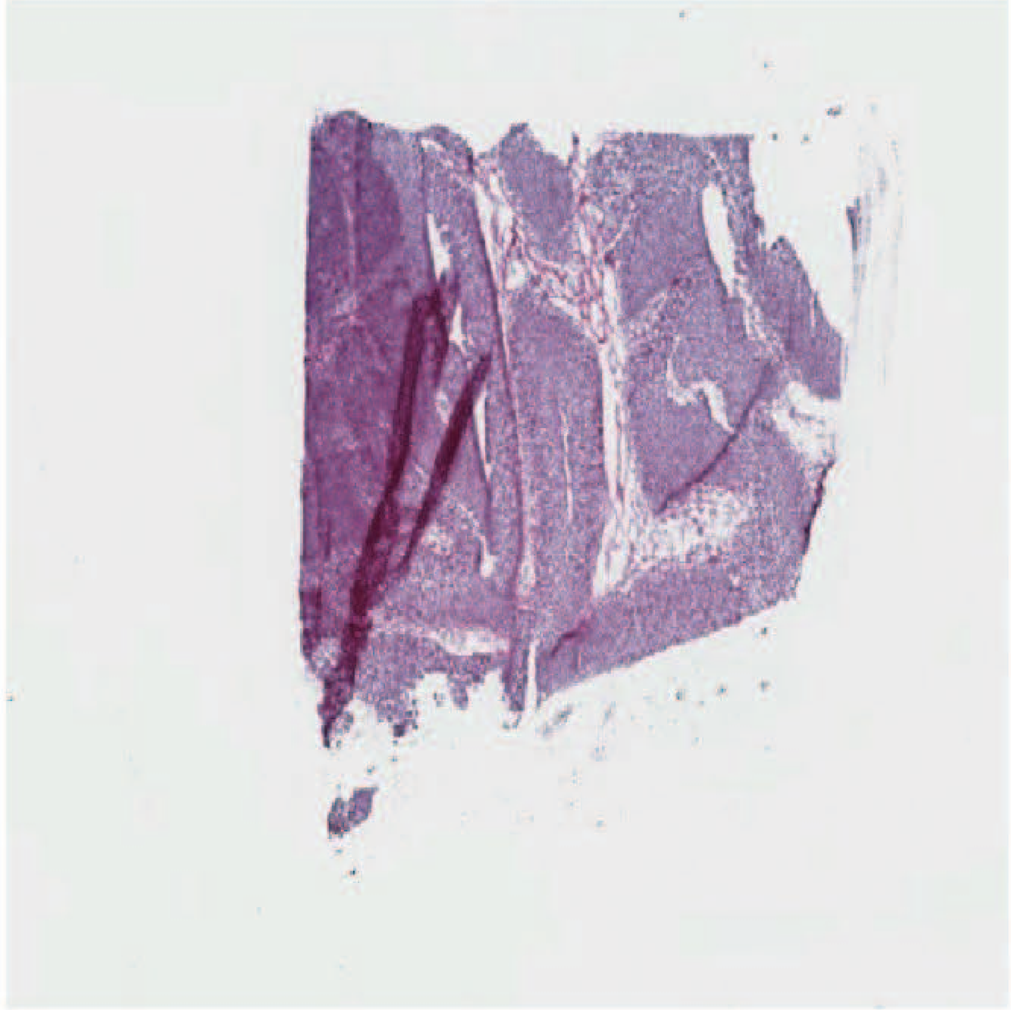

TCGA-ZF-AA4U Stromalscore： -2425.480112
