# Supplementary figures and images for: A high stroma-tumor ratio is associated with an immunosuppressive tumor microenvironment and a poor prognosis in bladder cancer
Source: Front Oncol. 2025 Aug 22;15:1604609. doi: 10.3389/fonc.2025.1604609 (PMC12411155; doi:10.3389/fonc.2025.1604609)

A

t-test result

|              | STR | N   | Avg.       | SD       | SE of mean |
|--------------|-----|-----|------------|----------|------------|
| Stromalscore | 0   | 282 | -1166.3234 | 691.5921 | 41.1837    |
|              | 1   | 125 | 404.8084   | 628.5658 | 56.2206    |

B

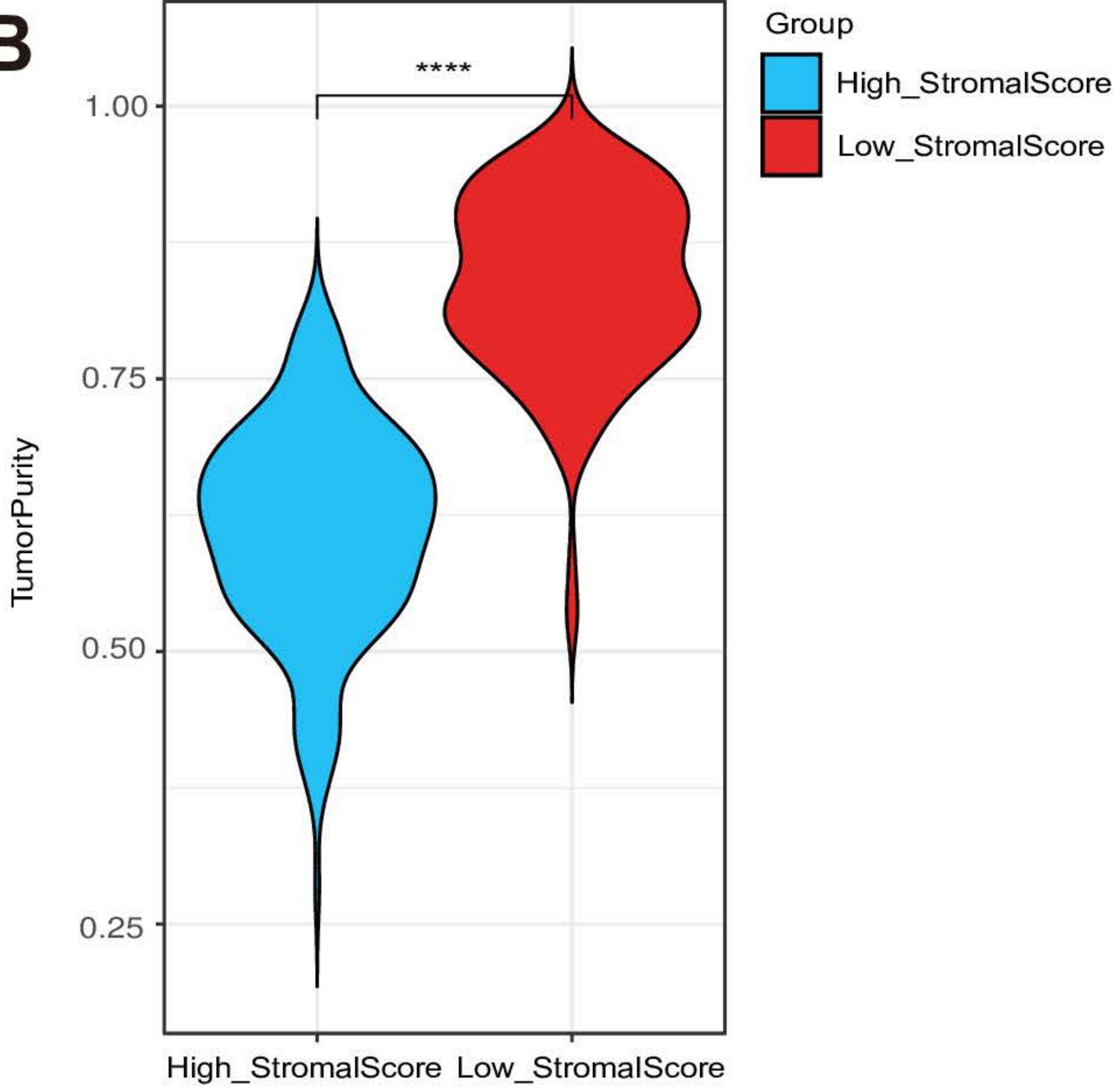

C

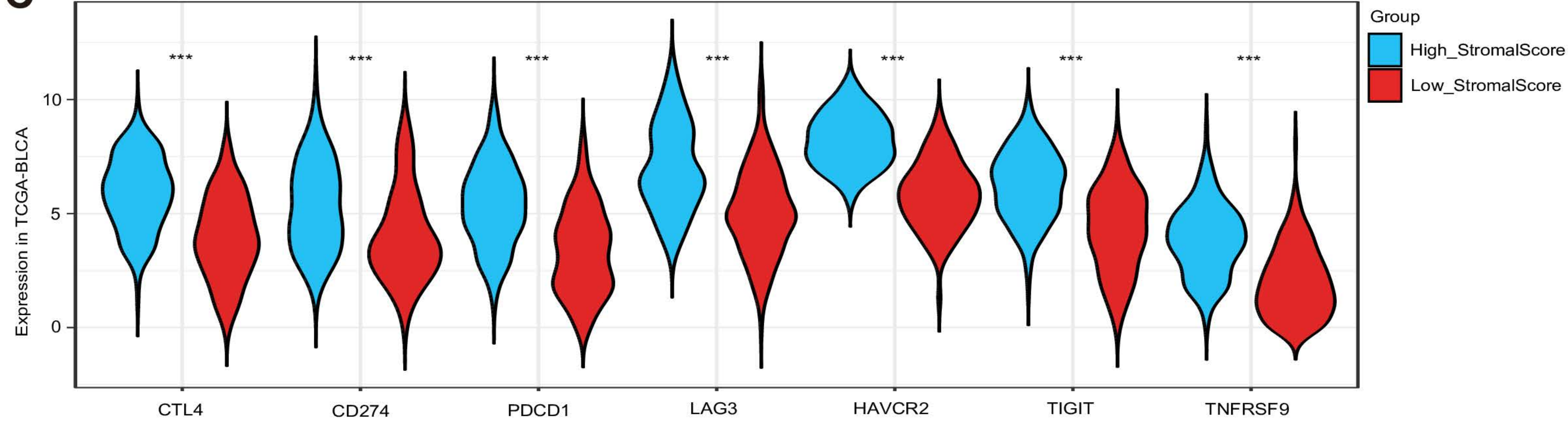

D

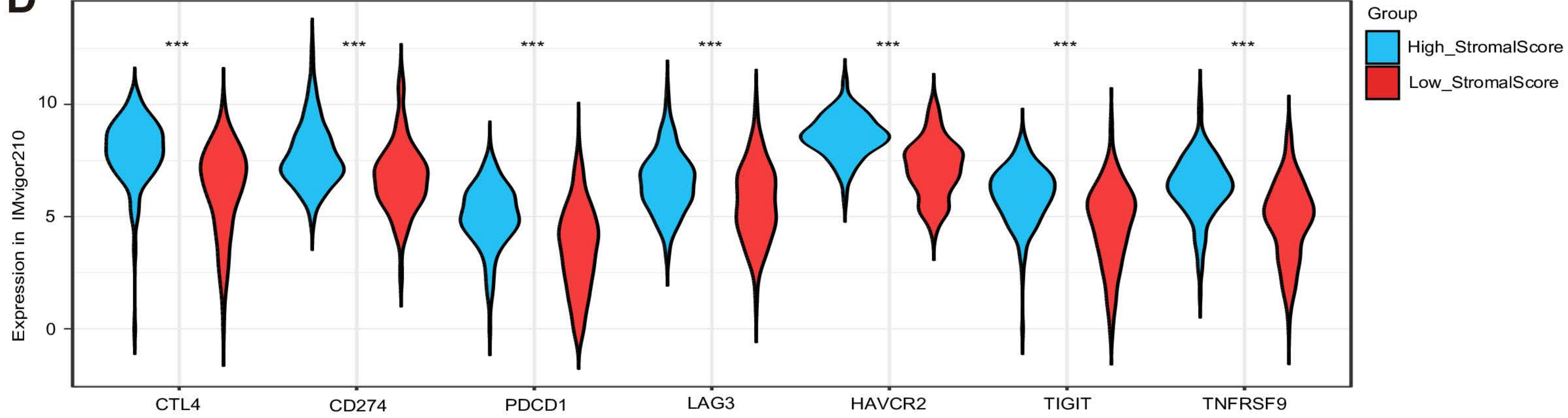

E

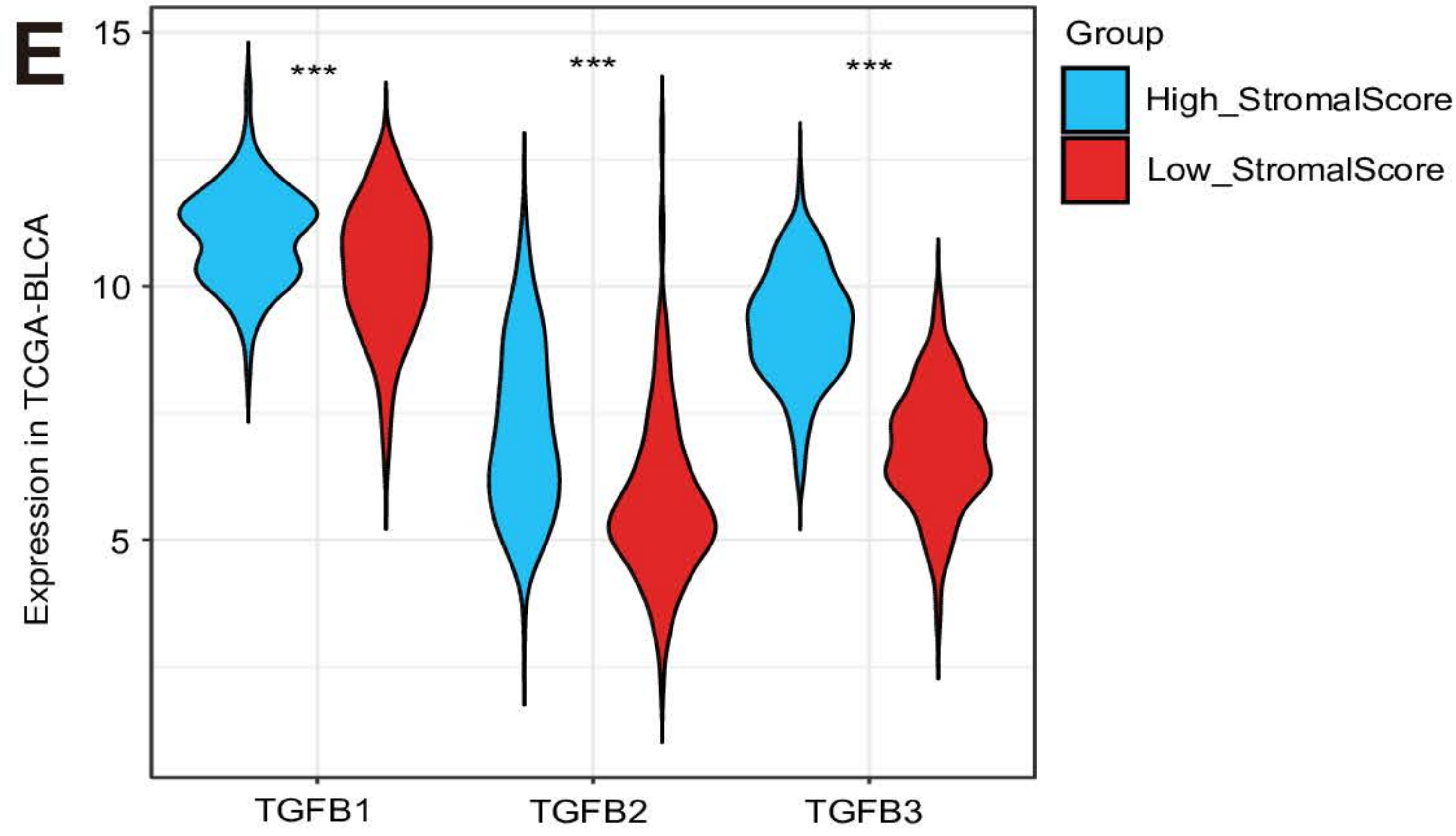

F

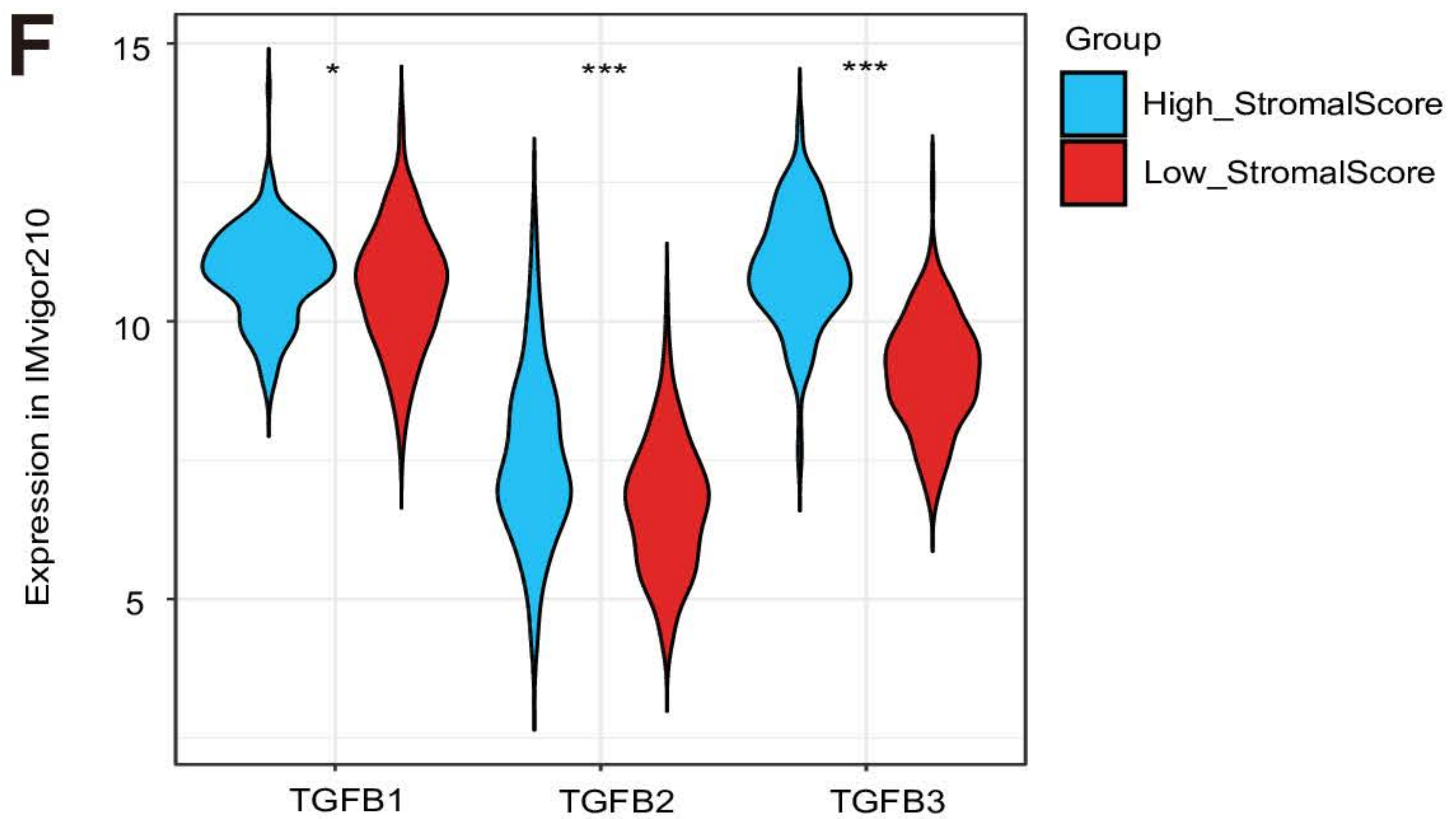

Supplement: Supplementary Figure 2 — High stroma microenvironment may lead to TME immune overdrive. (A) The t-test result between different STR subgroups. (B) Violin plot of tumor purity between subgroups with different stromal scores. (C, D) Expression levels of ICs of subgroups with different stromal scores in both TCGA-BLCA dataset and IMvigor210 dataset. (E, F) Expression of TGF-β signaling in both TCGA and IMvigor210 dataset. [file Image2.pdf]
